# Primate gastrulation and early organogenesis at single-cell resolution

**DOI:** 10.1038/s41586-022-05526-y

**Published:** 2022-12-14

**Authors:** Jinglei Zhai, Jing Guo, Haifeng Wan, Luqing Qi, Lizhong Liu, Zhenyu Xiao, Long Yan, Daniel A. Schmitz, Yanhong Xu, Dainan Yu, Xulun Wu, Wentao Zhao, Kunyuan Yu, Xiangxiang Jiang, Fan Guo, Jun Wu, Hongmei Wang

**Affiliations:** 1 State Key Laboratory of Stem Cell and Reproductive Biology, Institute of Zoology, Chinese Academy of Sciences, Beijing, China; 2 Institute for Stem Cell and Regeneration, Chinese Academy of Sciences, Beijing, China; 3 Beijing Institute for Stem Cell and Regenerative Medicine, Beijing, China; 4 University of Chinese Academy of Sciences, Beijing, China; 5 Department of Molecular Biology, University of Texas Southwestern Medical Center, Dallas, TX USA; 6 NHC Key Laboratory of Study on Abnormal Gametes and Reproductive Tract, Anhui Medical University, Hefei, China; 7 Department of Obstetrics and Gynecology, the First Affiliated Hospital of Anhui Medical University, Hefei, China; 8 Hamon Center for Regenerative Science and Medicine, University of Texas Southwestern Medical Center, Dallas, TX USA; 9 Cecil H. and Ida Green Center for Reproductive Biology Sciences, University of Texas Southwestern Medical Center, Dallas, TX USA

**Keywords:** Embryogenesis, Organogenesis, Embryology

## Abstract

Our understanding of human early development is severely hampered by limited access to embryonic tissues. Due to their close evolutionary relationship with humans, nonhuman primates are often used as surrogates to understand human development but currently suffer from a lack of in vivo datasets, especially from gastrulation to early organogenesis during which the major embryonic cell types are dynamically specified. To fill this gap, we collected six Carnegie stage 8–11 cynomolgus monkey (*Macaca fascicularis*) embryos and performed in-depth transcriptomic analyses of 56,636 single cells. Our analyses show transcriptomic features of major perigastrulation cell types, which help shed light on morphogenetic events including primitive streak development, somitogenesis, gut tube formation, neural tube patterning and neural crest differentiation in primates. In addition, comparative analyses with mouse embryos and human embryoids uncovered conserved and divergent features of perigastrulation development across species—for example, species-specific dependency on Hippo signalling during presomitic mesoderm differentiation—and provide an initial assessment of relevant stem cell models of human early organogenesis. This comprehensive single-cell transcriptome atlas not only fills the knowledge gap in the nonhuman primate research field but also serves as an invaluable resource for understanding human embryogenesis and developmental disorders.

## Abstract (web summary)

In-depth transcriptomic analyses of 56,636 single cells from monkey embryos revealed transcriptional features of major perigastrulation cell types, and comparative analyses with mouse embryos and human embryoids uncovered conserved and divergent features of perigastrulation development across species.

## Main

In humans, the developmental periods of gastrulation and early organogenesis largely remain a ‘black box’ due to limited access to research embryos. Recently, single-cell RNA sequencing (scRNA-seq) data from six aborted human embryos (one Carnegie stage^1^ (CS) 7 (ref. ^2^) and five CS12–16 (ref. ^3^)) and 15 CS3–7 cynomolgus monkey embryos^4^ became available, providing valuable resources for the study of primate early postimplantation development. Despite these advances, single-cell transcriptomes of CS8–11 human and nonhuman primate (NHP) embryos are still not available, which severely hinders the study of primate perigastrulation development and aetiology underlying several most common forms of congenital malformations. To fill this knowledge gap, we generated a comprehensive single-cell atlas of CS8–11 cynomolgus monkey (herein referred to as monkey) embryos and studied the major molecular and cellular processes during this critical developmental period in primates.

## A transcriptome atlas of monkey embryos

**Fig. 1:**
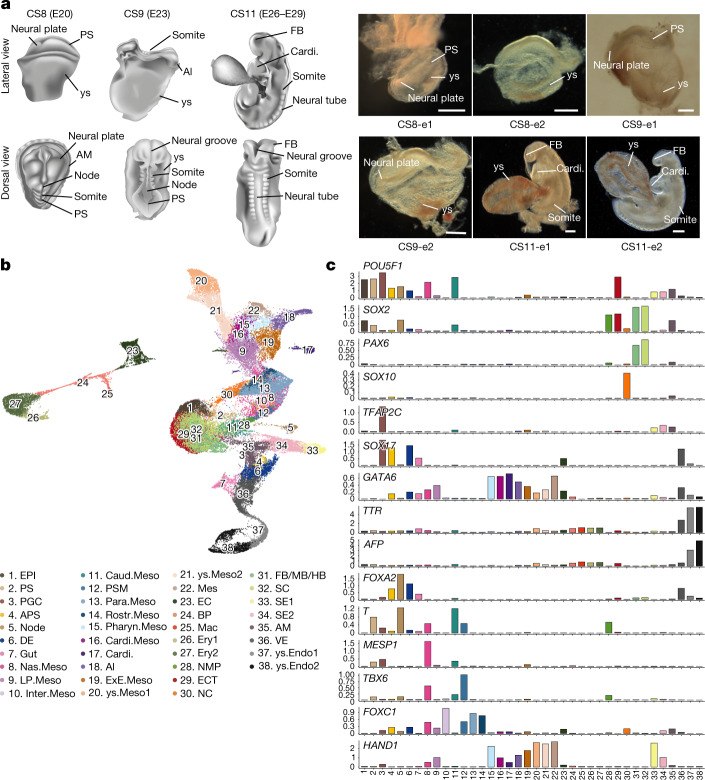
Construction of single-cell transcriptome atlas of cynomolgus monkey embryos at CS8–11. **a**, Left, flowchart overview of the dynamic morphogenesis of CS8–11 monkey embryos. ys, yolk sac; Al, allantois; AM, amnion; FB, forebrain; Cardi., cardiac. Right, representative bright-field images of embryos obtained from indicated developmental stages; e, embryo tissue; *n* = 2 for each stage sample. Scale bars, 200 µm. **b**, UMAP plot showing the 38 major cell types. EPI, epiblast; Mes, mesenchyme; EC, endothelial cell; BP, blood progenitor; Mac, macrophage; Ery, erythrocyte; ys.Endo, yolk sac endoderm. **c**, Bar charts showing the over-represented genes in each cell type. *POU5F1* expression is higher in pluripotent cells such as EPI and PGCs; *SOX2* shows higher expression in pluripotent cells, ECT and FB/MB/HB; *PAX6* is essential for neural development, and *SOX10* marks NC. *TFAP2C* is over-represented in PGCs, AM and SE, whereas *SOX17* expression is high in endodermal lineage and PGCs. *GATA6* is overexpressed in mesoderm and endodermal lineages. *TTR* and *AFP* expression levels are high in ys.Endo. *FOXA2* is highly expressed in node and endodermal lineage. *T* is over-represented in PS and mesodermal lineage. *MESP1* marks the Nas.Meso, and *TBX6* is over-represented in PS, Nas.Meso, NMP and PSM. *FOXC1* and *HAND1* show high expression in mesodermal lineage.

**Extended Data Fig. 1:**
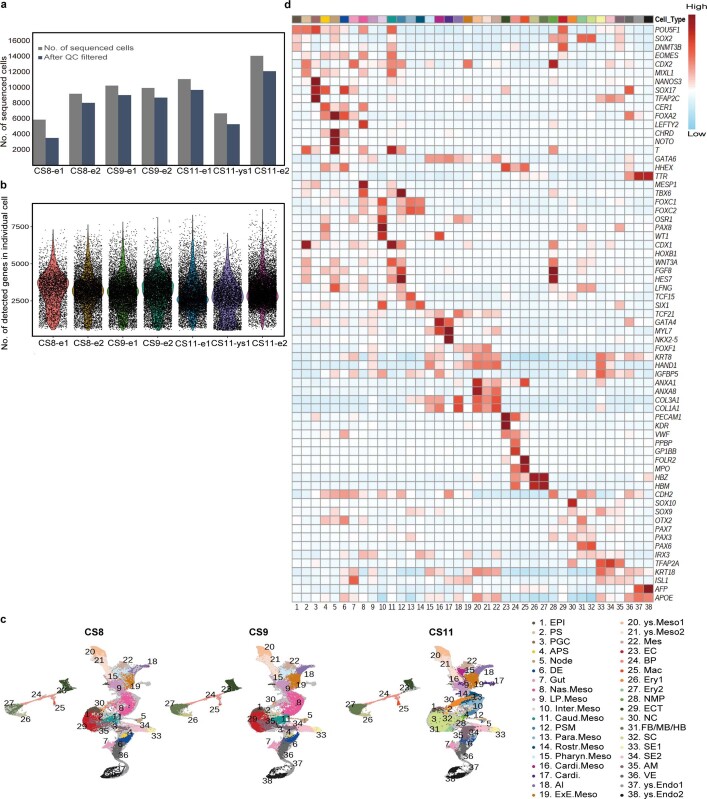
Transcriptomic features of the cynomolgus monkey embryos at CS8–11. **a**, Bar graph showing the number of collected single cells and filtered cells of each sample. **b**, Box plot of the gene numbers detected in each sample. **c**, UMAPs showing the distribution of 38 major cell types in indicated developmental stages (CS8–11). EPI, epiblast; PS, primitive streak; PGC, primordial germ cells; APS, anterior primitive streak; DE, definitive endoderm; Nas.Meso, nascent mesoderm; LP.Meso, lateral plate mesoderm; Inter.Meso, intermediate mesoderm; Caud.Meso, caudal mesoderm; PSM, presomitic mesoderm; Para.Meso, paraxial mesoderm; Rostr.Meso, rostral mesoderm; Pharyn.Meso, pharyngeal mesoderm; Cardi.Meso, cardiac mesoderm; Cardi., cardiac tissue; Al, allantois; ExE.Meso, extra-embryonic mesoderm; ys.Meso, yolk sac mesoderm; Mes, mesenchyme; EC, endothelial cell; BP, blood progenitor; Mac, macrophage; Ery, erythrocytes; NMP, neuromesodermal progenitor; ECT, ectoderm; NC, neural crest; FB/MB/HB, forebrain/midbrain/hindbrain; SC, spinal cord; SE, surface ectoderm; AM, amnion; VE, visceral endoderm; ys.Endo, yolk sac endoderm. **d**, Heatmap visualizing the representative differentially expressed genes (DEG, here and after) for the 38 cell clusters. Colors from blue to red indicate the low to high expression of different genes in indicated cell types, here and after.

We collected six monkey embryos during embryonic day (E) 20–29, which were staged at CS8, CS9 and CS11 (Fig. 1a and Supplementary Table 1). All embryos appeared morphologically normal with expected anatomical features—for example, primitive streak (PS) and enlarged yolk sac in CS8 and CS9 embryos and forebrain, cardiac structure and somites in CS11 embryos. Samples were dissociated into single cells, with 67,418 sequenced using the 10X Genomics Chromium platform (Supplementary Table 1). After filtering out doublets/multiplets and low-quality cells (fewer than 500 genes detected), a total of 56,636 cells was retained for subsequent analyses with a median of 3,017 genes detected per cell (Extended Data Fig. 1a,b and Supplementary Tables 1 and 2). Based on the expression of known lineage markers and comparison with datasets from mouse embryos at corresponding developmental stages^5–7^, 38 major clusters were identified (Fig. 1b,c, Extended Data Fig. 1c,d and Supplementary Table 3). The epiblast (EPI) and PS cells (cluster nos. 1 and 2) identified from CS11 embryos were greatly under-represented, suggesting that gastrulation was nearing completion at this developmental stage (Fig. 1b, Extended Data Fig. 1c and Supplementary Table 2).

## Development landscape of primitive streak

**Fig. 2:**
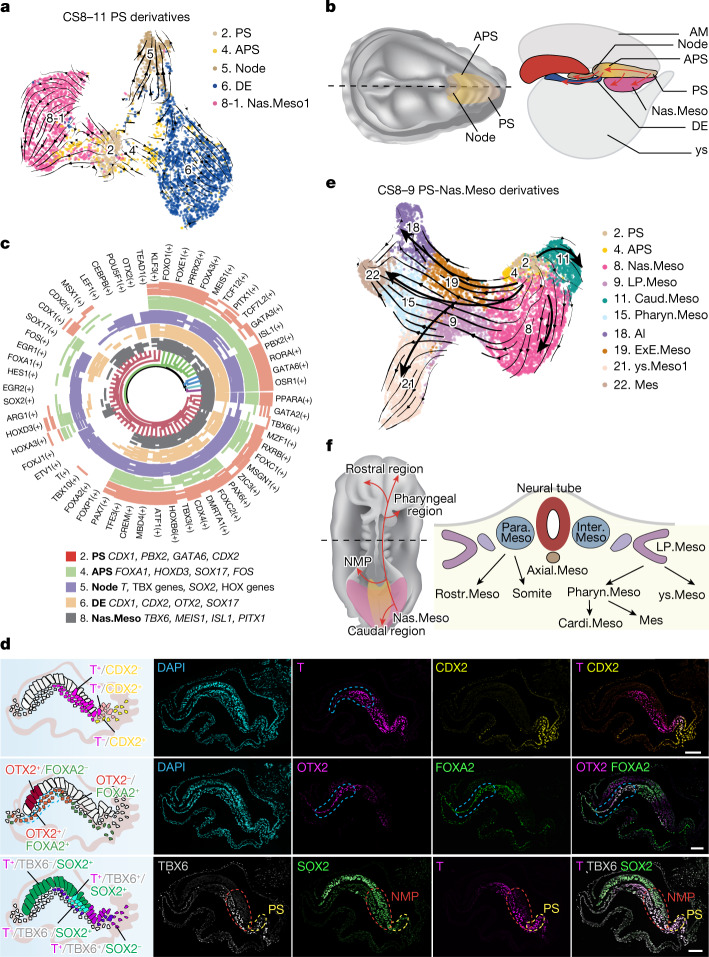
Developmental trajectories of PS and its derivatives from monkey embryos. **a**, UMAP showing PS and its derivatives from CS8–11 monkey embryos with trajectories inferred from RNA velocity analysis. Black arrows represent calculated velocity trajectories. **b**, Schematic diagram showing a CS8 embryo. Left, dorsal view; right, lateral view showing potential trajectories from PS to APS, DE, node and Nas.Meso. **c**, Joint hierarchical clustering (HC) showing the circular heatmap with representative TFs upregulated in PS (red), APS (green), node (purple), DE (orange) and Nas.Meso (grey). Genes marked with the same colour by HC show similar expression patterns in different cell types. **d**, Left, diagrams summarizing the distribution of indicated cell types in the region of interest, based on IF images on the right. Right, IF results showing localization of T^+^, CDX2^+^, OTX2^+^, FOXA2^+^, TBX6^+^ and SOX2^+^ cells in a monkey E22 embryo. T, marker for PS and some mesoderm; CDX2, marker for PS; OTX2, marker for visceral endoderm, definitive endoderm, neural ectoderm and some mesoderm; FOXA2, marker for endoderm and some mesoderm; TBX6, marker for PS, NMP and some mesoderm; SOX2, marker for ECT. DAPI, marker for DNA, here and after. Blue dashed lines indicate potential organizer location; yellow and red dashed lines denote PS and NMP regions, respectively. Scale bars, 100 µm. **e**, RNA velocity overlaid on UMAP coloured by PS and mesodermal fates from CS8–9 embryos. **f**, Left, schematic diagram showing the potential migration and differentiation route of Nas.Meso from the dorsal view of a monkey CS9 embryo. Right, diagram showing mesodermal derivatives from a transverse section.

**Extended Data Fig. 2:**
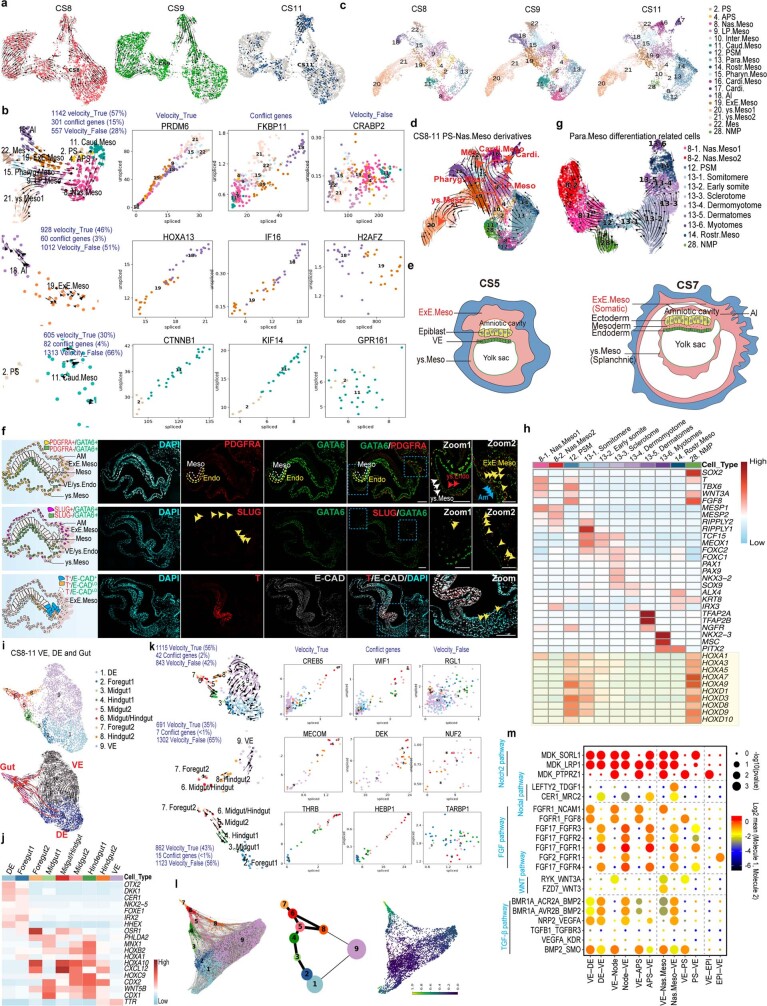
Transcriptomic characteristics of monkey primitive streak and mesoendoderm. **a**, UMAPs overlaid with RNA velocity of PS derivatives from embryos at indicated stages. Black arrows flowed hereafter represent calculated velocity trajectories. **b**, (left) UMAPs overlaid with RNA velocity of PS-mesoderm lineage cells after micro-clustering (see Methods). (right) Phase portraits of representative Velocity_True, Conflict and Velocity_False genes (unspliced versus spliced dependency) that support these velocity analyses. Each point corresponds to a “pseudo” cell, colored by cluster labels, here and after. **c**, UMAPs showing the distribution of PS-mesoderm lineage derivatives at indicated developmental stages. **d**, UMAP overlaid with RNA velocity of PS-mesoderm lineage derivatives from CS8–11 embryos. **e**, Diagrams showing potential originations of extra-embryonic mesenchymal cells (EXMCs, including ys.Meso, ExE.Meso, some mesenchyme, among others) at pre (CS5) or perigastrulation (CS7) stage. The left diagram indicating the EXMCs at pregastrulation are putatively generated from hypoblast derived yolk sac. The right one indicating the putative splanchnic or somatic migrating route of different EXMCs generated from PS/mesoderm at perigastrulation stage. **f**, (left) Diagrams summarizing the distribution of indicated cell types in the region of interest, based on the right IF images. (right) IF results exhibiting the localization of PDGFRA^+^, GATA6^+^, SLUG^+^, T^+^, and E-CAD^low^ cells on a CS8 (E22) monkey embryo. PDGFRA, the marker for mesoderm. GATA6, the marker for EXMCs, endoderm and some mesoderm. T, the marker for primitive streak and some mesoderm. SLUG, the marker for epithelial-mesenchymal transition. E-CAD, the marker for epithelial cells. DAPI, marker for DNA, here and after. Blue dashed frames refer to the zoomed regions; yellow arrowheads indicating the potential ExE.Meso; Blue arrowheads indicating the AM. White arrowheads indicating the ys.Meso (PDGFRA^+^/GATA6^+^) and red arrowheads indicating the ys.Endo (PDGFRA^−^/GATA6^+^) of the CS8 secondary yolk sac. Scale bars, 100 µm. **g**, UMAP overlaid with RNA velocity of Para.Meso differentiation related cell types from CS8–11 embryos. **h**, Heatmap showing the Para.Meso-related cell types based on DEG analysis. Color bar in heatmap, normalized expression level of different genes in indicated cell types, here and after. **i**, (top) UMAP showing the subtypes of VE, DE and Gut from CS8–11 embryos. (bottom) UMAP overlaid with velocity of VE, DE and Gut cells. **j**, Heatmap showing the representative DEGs of different gut tube subtypes in **i**. **k**, (left) UMAPs overlaid with RNA velocity of endoderm lineage cells after micro-clustering. (right) Phase portraits of representative Velocity_True, Conflict and Velocity_False genes that support the velocity analyses. **l**, (left) FA1 (Force-directed graph) exhibiting expected descendant and ancestor relationship between different cell types. (middle) PAGA analysis on DE, VE and different Gut cells. The boldness of the line indicates the degree of the relationship between clusters. Cell type annotations are color coded. (right) FA1 map showing the diffusion pseudotime analysis of the 9 annotated cell types. The color gradient from dark to bright refers to the level of pseudotime differentiation diffusion from low to high. **m**, Overview of the representative ligand-receptor interactions between VE and embryonic cells including EPI, ECT, PS, APS, Nas.Meso, and DE, analyzed by Cellphone DB. On the horizontal axis, the bottom factors indicating the ligand, and the top factors indicating the receptors. On the vertical axis, the left one indicating the tissue secreting signal-ligands, while the right one indicating the tissue expressing the receptors and receiving the signals. The *P* value (indicated by circle size, scale on the right) in the analyses indicates the likelihood of cell-type enrichment of each ligand-receptor complex, here and after. The means of the average expression level of interacting molecule 1 in cluster 1 and interacting molecule 2 in cluster 2 are indicated by color.

To study the molecular and cellular dynamics during monkey gastrulation and early organogenesis, we used RNA velocity, which predicts differentiation trajectories by leveraging splicing kinetics^8^. We first focused on PS formation-related clusters, which include PS (no. 2), anterior primitive streak (APS, no. 4), definitive endoderm (DE, no. 6), node (no. 5) and nascent mesoderm (Nas.Meso, no. 8) clusters (Fig. 1b). Similar to that in mice^7^, RNA velocity predicted a trifurcating differentiation trajectory of monkey PS/APS towards DE, Nas.Meso and node as gastrulation advances (Fig. 2a,b and Extended Data Fig. 2a). Single-cell regulatory network inference and clustering (SCENIC) and immunofluorescence (IF) analyses showed that several transcription factors (TFs) were enriched in clusters PS (for example, *GATA6* and *PBX2*), APS (for example, *FOXA1* and *HOXD3*), Nas.Meso (for example, *TBX6* and *MEIS1*), DE (for example, *CDX1* and *OTX2*) and node (for example, *TBX* and *HOX*) (Fig. 2c,d), suggesting their roles in different steps of monkey PS formation. Consistent with a study in mice^9^, differentially expressed gene (DEG) and IF analyses provided support that FOXA2^+^ cells putatively contributed to DE in monkeys (Extended Data Fig. 1d and Fig. 2d).

Nas.Meso gives rise to most mesoderm cell types. Based on DEG analysis, Nas.Meso derivatives, including intermediate (Inter.Meso), paraxial (Para.Meso), rostral (Rostr.Meso), pharyngeal (Pharyn.Meso), cardiac (Cardi.Meso), lateral plate (LP.Meso), caudal (Caud.Meso) mesoderm cells and extra-embryonic mesenchymal cells (EXMCs, including allantois (Al), yolk sac (ys.Meso) and extra-embryonic mesoderm cells (ExE.Meso)), were identified (Fig. 2e, Extended Data Figs. 1d and 2b,c and Supplementary Table 3). Cardi.Meso, Inter.Meso, Rostr.Meso, neuromesodermal progenitor (NMP) and presomitic mesoderm (PSM) cells did not manifest as distinct clusters until CS11, when the numbers of cells identified as Nas.Meso and Caud.Meso were greatly reduced (Extended Data Fig. 2c). To gain insight into early mesoderm differentiation, we generated RNA velocity maps of PS and mesoderm cells, which predicted differentiation trajectories of Nas.Meso towards LP.Meso, ExE.Meso and Para.Meso, followed by LP.Meso to ys.Meso1 and Pharyn.Meso (Fig. 2e,f and Extended Data Fig. 2b,d), consistent with studies in chicks and mice^10^. It was hypothesized that early primate EXMCs, which putatively originated from hypoblast-derived primary yolk sac before gastrulation, subsequently merged with PS-derived EXMCs to establish the allantoic stalk^11–14^ (Extended Data Fig. 2e). Our RNA velocity and IF analyses support the potential contribution of PS (Nas.Meso) towards EXMCs (ExE.Meso, ys. Meso and Al) during CS8–11 in primates^12^ (Fig. 2e and Extended Data Fig. 2b,d,f).

Somitogenesis, the process initiated from a subtype of NMP, has been extensively studied in mice^15^ but not in primates. To better understand primate somitogenesis, we performed IF analysis and identified two putative NMP populations in a CS8 embryo: SOX2^low^/T^high^/TBX6^high^ (contributing to somite) and SOX2^high^/T^low^/TBX6^low^ (contributing to spinal cord (SC)) (Fig. 2d). Based on expression patterns of signalling pathway components and regional markers^16^, we identified Rostr.Meso (*PITX2*, *IRX3*) and several somitic cell types in the Para.Meso cluster, which contained somitomere (also called segmentation boundary, *RIPPLY1/2*), early somite (*TCF15*, *FOXC2*, *MEOX1*), sclerotome (*PAX1*, *PAX9*, *NKX3.2*, *SOX9*) and dermomyotome (*PAX7*, *ALX4*, *TFAP2A*) (Extended Data Fig. 2g,h). RNA velocity analysis on NMP, PSM and Para.Meso clusters further revealed their putative lineage relationships (Extended Data Fig. 2g).

To gain insight into gut tube (GT) formation in primates, we focused on DE, visceral endoderm (VE) and gut clusters. Our analyses identified seven subclusters of foregut, midgut and hindgut cells (Extended Data Fig. 2i–k). Foregut contained cells expressing *HHEX* (Foregut1) and *PHLDA2* (Foregut2), Midgut cells were separated into Midgut1 (*MNX1*), Midgut2 (*HOXB2*, *HOXC9*) and Midgut/Hindgut (*HOXA10*, *CXCL12*), whereas Hindgut cells included subclusters Hindgut1 (*HOXA10*) and Hindgut2 (*CDX2*) (Extended Data Fig. 2j). To help identify the origin(s) of gut cells in primates, we performed RNA velocity analysis which predicted: (1) Foregut1 was solely derived from DE; and (2) Hindgut2 was exclusively contributed by VE whereas Foregut2, Midgut1/2, Midgut/Hindgut and Hindgut1 clusters contained both DE (mostly) and VE cells (Extended Data Fig. 2i,k). These RNA velocity predictions were further validated by transport map, partition-based graph abstraction (PAGA) and pseudotime analyses (Extended Data Fig. 2l), consistent with the DE and VE dual origins of gut cells in mice^6,17^. VE, through secreted inhibitors of WNT and NODAL pathways, plays an important role in anterior patterning of mouse epiblast (EPI)^18,19^. We performed CellPhoneDB analysis and identified several conserved ligand–receptor interactions of TGF-β (BMP, NODAL), WNT and FGF pathways between VE and EPI/EPI derivatives (PS, APS, DE, node and Nas.Meso)^20^. Interestingly, interactions mediated by ligand–receptor pairs of the Notch2 pathway were over-represented between monkey EPI derivatives and VE whereas mouse embryos with perturbed Notch signalling developed normally beyond gastrulation^21^, implying a new role of Notch2 signalling during primate gastrulation. Furthermore, more ligand–receptor interactions were identified between VE and EPI derivatives than between VE and EPI, suggesting dynamic communications between extra-embryonic and embryonic cells during gastrulation (Extended Data Fig. 2m).

Taken together, these analyses identified major cell types during monkey PS development, early mesoderm and endoderm differentiation and shed light on somitogenesis and GT formation in primates.

## Developmental landscape of ectoderm

**Extended Data Fig. 3:**
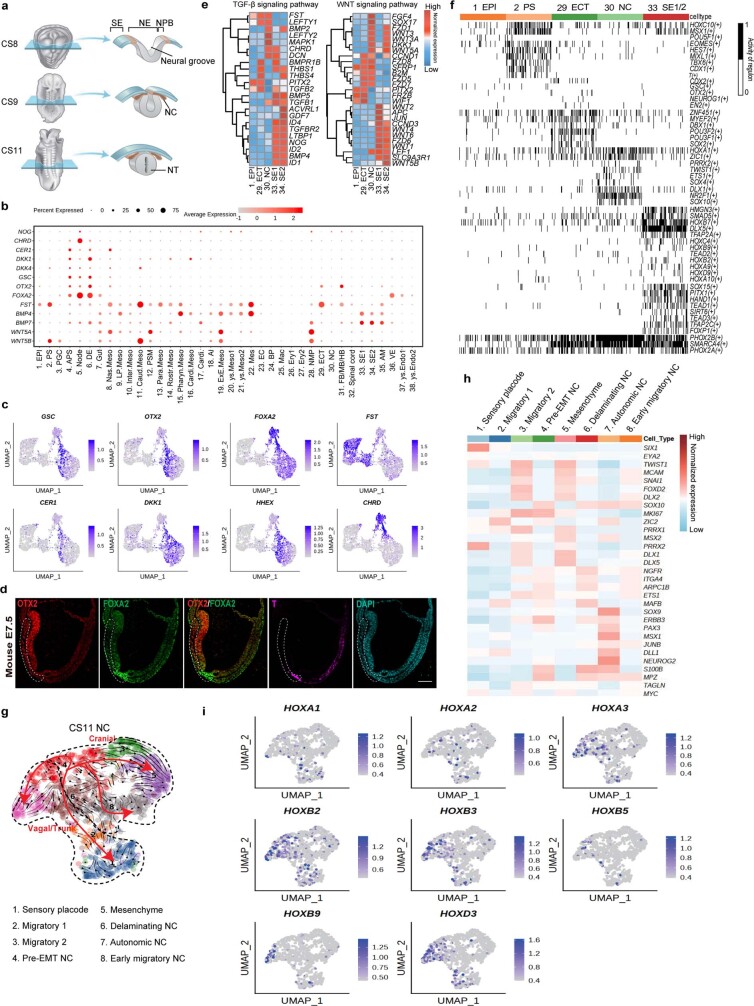
The single-cell transcriptomic analysis on ectoderm. **a**, Diagram summarizing the formation of neural tube and the differentiation of neural and non-neural ectoderm in the CS8–11 primate embryos. NPB, neural plate border; NT, neural tube. **b**, Dot plot map showing the expression levels of specific marker genes of the potential organizer. The plot sizes from 0–75 indicate the percentage of cells expressed the indicated genes. Colors from grey to red indicate the average expression level of different genes in indicated cell types. **c**, UMAPs showing the expression of organizer marker genes in PS, node, DE, Nas.Meso, which are particularly analyzed in Fig. 2a. Color bars in UMAPs, normalized expression level of indicated genes from high (purple) to low (grey), here and after. **d**, Representative IF results exhibiting the localization of organizer (OTX2^+^/FOXA2^+^/T^+/Low^) in E7.5 mouse embryos. White dashed lines highlight the potential region of organizer cells. DAPI, marker for DNA. Scale bar, 100 µm. n = 3 biologically independent samples. **e**, Heatmaps showing the expression of genes in TGF-β and WNT signalling pathways in relative cell types. Color bar in heatmap, the normalized expression level of different genes in indicated cell types, here and after. **f**, Heatmap showing the representative transcription factors (TFs) in the differentiation of ECT. Color bar, activity of regulons, indicating the active (black, 1) or non-active (white, 0) of different transcriptome factors. **g**, UMAP overlaid with the velocity analysis on different NC subtypes. EMT, epithelial-mesenchymal transition. **h**, Heatmap showing the representative DEGs in various NC subtypes. **i**, UMAPs showing the expression patterns of HOX family genes in NC.

After definitive endoderm and embryonic mesoderm are formed, the remaining epiblast cells become the ectoderm (ECT, cluster no. 29), giving rise to centrally located neural ectoderm (NE, also called neural plate), surface ectoderm (SE, cluster nos. 33 and 34) at the periphery and neural plate border (NPB) between the two, which ventrally delaminates and differentiates into neural crest (NC, cluster no. 30) (Fig. 1b and Extended Data Fig. 3a). The neural plate then thickens, bends and folds to form the neural tube, the precursor of the central nervous system (CNS)^22^ (Extended Data Fig 3a).

Specification of NE and SE along the mediolateral axis in zebrafish, chick, mouse embryos and human embryonic stem cell (hESC) derivatives depends on BMP and WNT gradients generated by the Spemann–Mangold organizer—a group of cells that plays a key role in the establishment of dorsal–ventral (D–V) and anterior–posterior (A–P) axes during gastrulation^22–27^. We found that organizer-related genes^27^, including *GSC*, *OTX2*, *FOXA2*, *FST*, *CER1*, *DKK1*, *HHEX* and *CHRD*, were highly expressed in some cells from PS, APS, DE and node clusters (Extended Data Fig. 3b,c). IF analyses of OTX2, FOXA2 and T helped localize putative organizer cells to the anterior region beneath the ectoderm in a CS8 monkey embryo (Fig. 2d), which is consistent with mouse organizer cells in the E7.5 mouse embryo (Extended Data Fig. 3d). In addition, many genes related to TGF-β and WNT pathways were found upregulated in SE but not in NE, suggesting selective activation of these pathways during SE differentiation (Extended Data Fig. 3e). SCENIC analysis further showed elevated expression of TFs including *SOX2*, *POU3F2*, *EN2*, *OTX2* and *NEURUG1* in ECT, and *TFAP2A*, *TFAP2C*, *DLX5* and *HOX* family genes in SE (Extended Data Fig. 3f), which may help specify and/or stabilize their lineage identities.

Neural crest is a transient, multipotent and migratory cell population^22,28^. Notably, the number of cells expressing NC specification genes (for example, *SOX10*, *SOX9*, *PAX3*, *FOXD3* and *SNAI2*) greatly increased in CS11 embryos (Extended Data Fig. 3g,h). We identified eight subpopulations of NC cells from CS11 embryos, which include pre-EMT (*PAX3*, *ZIC2*), delaminating (*MAFB*, *MEF2C*), early migratory (*SNAI2*, *FOXD2*), migratory1/2 (*TWIST1*, *MCAM*), mesenchymal (*PRRX1*), sensory (*SIX1*, *EYA2*) and autonomic (*S100B*, *MPZ*) cells (Extended Data Fig. 3g,h). The expression patterns of *HOX* genes helped distinguish cranial and vagal/trunk NC subtypes (Extended Data Fig. 3g,i). RNA velocity analysis further predicted that (1) pre-EMT and delaminating NCs gave rise to migrating progenitors that ultimately contributed to both cranial and vagal/trunk NC subtypes; and (2) cranial, but not vagal/trunk, NC contributed to mesenchymal cells (Extended Data Fig. 3g). These predictions are consistent with mouse studies^28,29^.

**Fig. 3:**
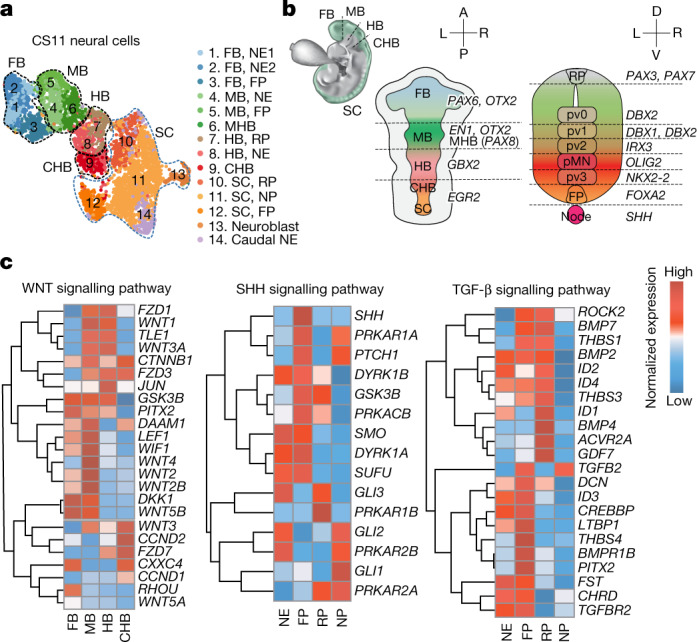
Developmental landscape of neural cells from monkey embryos. **a**, UMAP plot showing subtypes of neural cells (including FB/MB/HB and SC cells) from CS11 embryos. **b**, Schematics showing regionalized expression patterns of different brain regions (upper left) along A–P (middle) and D–V axes (right). **c**, Heatmaps showing expression of genes in WNT, SHH and TGF-β signalling pathways in indicated neural cells along the A–P and D–V axes.

**Extended Data Fig. 4:**
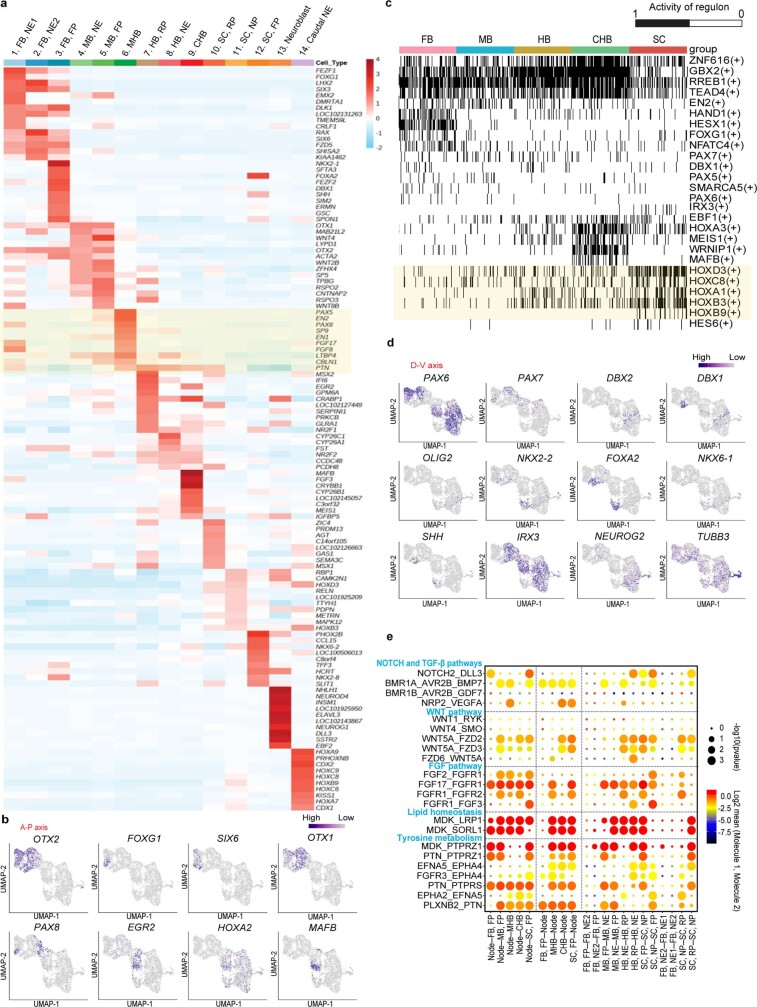
The transcriptomic features of different neural cells. **a**, Heatmap showing the representative DEGs in various neural cells from CS11 embryos. MHB, midbrain and hindbrain boundary; CHB, caudal hindbrain; NE, neural ectoderm (in unclosed neural tube); NP, neural progenitor (in closed neural tube); FP, floor plate; RP, roof plate. Color bar, normalized expression level of different genes in indicated cell types. **b**, UMAPs showing the expression pattern of marker genes along the A-P axis. Color bar in UMAPs, normalized expression level of indicated genes from high (purple) to low (grey), here and after. **c**, Heatmap showing the representative TFs in different brain regions and spinal cord (SC) along the A-P axis. Color bar, activity of regulons, indicating the active (black, 1) or non-active (white, 0) of different TFs. **d**, UMAPs showing the expression pattern of marker genes along the D-V axis of closed or unclosed neural tube. **e**, Overview of the representative ligand-receptor interactions between node and different neural cells analyzed by Cellphone DB.

To gain insight into neural tube development in primates, we reanalysed the forebrain/midbrain/hindbrain (FB/MB/HB, cluster no. 31) and SC (cluster no. 32) cells from two CS11 embryos (Fig. 1b). Based on the expression patterns of *OTX2*, *EN1*, *EGR2* and *HOXA2,* among others, FB, MB, HB and SC cells along the A–P axis could be annotated (Fig. 3a,b, and Extended Data Fig. 4a,b). In addition, midbrain–hindbrain boundary (MHB), which is derived from MB and characterized by morphological constriction of the neural tube, could also be identified based on the upregulation of *PAX8*, *FGF8* and *PAX5* (Fig. 3a,b and Extended Data Fig. 4a,b). Next we focused on the WNT pathway and HOX family genes, which are known to regulate neural tube patterning along the A–P axis in other vertebrates^26,30^. Compared with FB, many WNT pathway-related genes were upregulated in MB cells, implying increased WNT activity (Fig. 3c). Many *HOX* genes were found enriched in neural cells from the trunk region, suggesting their roles in A–P patterning of neural tube (Extended Data Fig. 4c). Besides, we identified several specific TFs of FB (for example, *HAND1*, *HESX1*, *FOXG1*, *NFATC4*) and caudal hindbrain (CHB; for example, *HOXA3*, *MEIS1*, *WRNIP1*, *MAFB*) (Extended Data Fig. 4c).

To show the transcriptomic features of monkey neural tube along the D–V axis, we studied roof plate (RP), NE and floor plate (FP) cells identified by expression patterns of genes including *PAX7*, *PAX6* and *FOXA2* (ref. ^31^) (Fig. 3a,b and Extended Data Fig. 4a,d). Neural progenitor (NP) of ventral interneurons (pv, *DBX1*, *DBX2*, *NKX2-2*) and motor neurons (pMN, *OLIG2*) that regionalized along the D–V axis, and neuroblasts (*TUBB3*, *NEURODG2*), could be annotated in the spinal cord, suggesting that the neural tube was closed here in CS11 embryos (Extended Data Fig. 4d). In mice, D–V patterning of the neural tube is induced by opposing morphogens SHH from notochord (ventral) and TGF-β superfamily from epidermis (dorsal)^32,33^. Next, we studied gene expression patterns in SHH and TGF-β signalling pathways^33^ (Fig. 3c). We found that FP highly expressed *GSK3B*, *PTCH1* and *SHH* whereas RP upregulated the expression of *BMP2*, *BMP4* and *BMP7* (Fig. 3c). From ventral to dorsal, *GLI1* and *GLI2* were upregulated in NP whereas *GLI3* expression was specifically elevated in RP. Notably, in the unclosed part of neural tube, NE simultaneously expressed *GLI2* and *GLI3* but not *GLI1*, which is required for the regulation of pv3 specification^33,34^ (Fig. 3c). CellPhoneDB analysis further indicated a prodigious number of ligand–receptor interactions among different neural cells and their neighbours (Extended Data Fig. 4e).

Taken together, these findings show that NE and SE in monkeys were specified along the mediolateral axis at CS8, followed by NC differentiation at CS11. In addition, as the extension of the body plan took place, spatial organization of CNS divisions was orchestrated along the A–P and D–V axes.

## Cross-species comparison

**Fig. 4:**
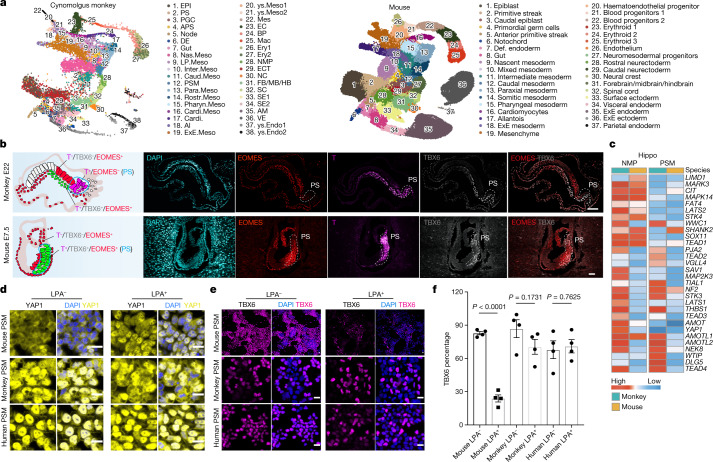
Cross-species transcriptomic comparison of early embryonic organogenesis. **a**, UMAPs showing the integrated datasets of CS8–11 monkey embryos (left) and E6.5–8.5 mouse embryos (right)^6^. **b**, Left, diagrams summarizing localization of EOMES^+^, T^+^ and TBX6^+^ cells in monkey E22 (top) and mouse E7.5 embryos (bottom). Blue lines circle the PS region. Right, IF results showing differences in EOMES expression of PS in monkey E22 embryo (top, cells coloured magenta, T^+^/EOMES^−^/TBX6^+/low^) and mouse E7.5 embryo (bottom, cells coloured green, T^+^/EOMES^+^/TBX6^+^). White dashed lines delineate the PS region. Scale bars, 100 μm. **c**, Heatmap showing the different expression patterns of notable molecules on the Hippo signalling pathway between monkeys and mice in terms of NMP and PSM cells. **d**, Different Hippo activities (YAP1) between mouse and primate (monkey and human) PSM-like cells, indicating species-specific requirement for Hippo activation during somitogenesis. Inhibition of Hippo activity leads to nuclear accumulation of YAP1 in mouse PSM-like cells. LPA is a Hippo inhibitor. Scale bars, 20 μm. Experiment was repeated independently three times. **e**, Inhibition of Hippo activity impairs differentiation of mouse PSM-like cells but shows only moderate effect on monkey PSM-like cell differentiation. Scale bars, 20 µm. Experiments were repeated independently three times. **f**, Quantification of percentage of TBX6^+^ from total cells; total cell number was calculated by counting the number of nuclei (DAPI). Data shown as mean ± s.d. (*n* = 4 biological replicates; experiments were independently repeated three times and quantified twice, with similar results). *P* values were determined by unpaired two-tailed *t*-test. Mouse LPA^−^ group versus LPA^+^ group, *P* < 0.0001; monkey LPA^−^ group versus LPA^+^ group, *P* = 0.173102; human LPA^−^ group versus LPA^+^ group, *P* = 0.762472. Source data

**Extended Data Fig. 5:**
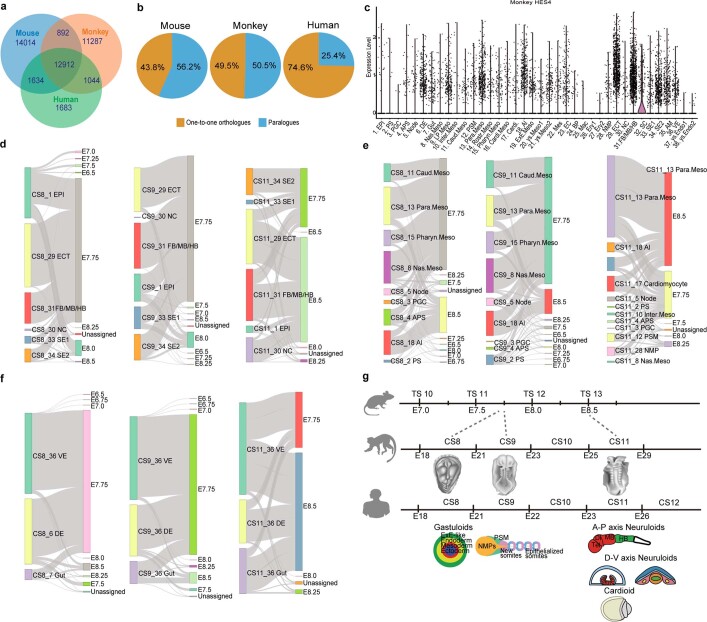
The cross-species comparison on orthologues and developmental stages. **a**, The number of orthologues and paralogues between mouse, monkey and human. In total 26135 genes from macaque embryonic dataset (orange), 29452 genes from mouse embryonic dataset (blue), and 17273 genes from human embryonic dataset (green) (CS7 and CS12) are included in this analysis. **b**, The percentage of one-to-one orthologues in each species. **c**, The expression of HES4 in different cell types in monkey embryos. **d**–**f**, Curve graphs showing the comparable developmental stage between monkey and mouse of different cell types from ectoderm (**d**), mesoderm and PGC (**e**), and endoderm (**f**). **g**, Schematic summarizing the cross-species transcriptomic and developmental stage comparison between mouse and monkey natural embryos and human embryoids.

**Extended Data Fig. 6:**
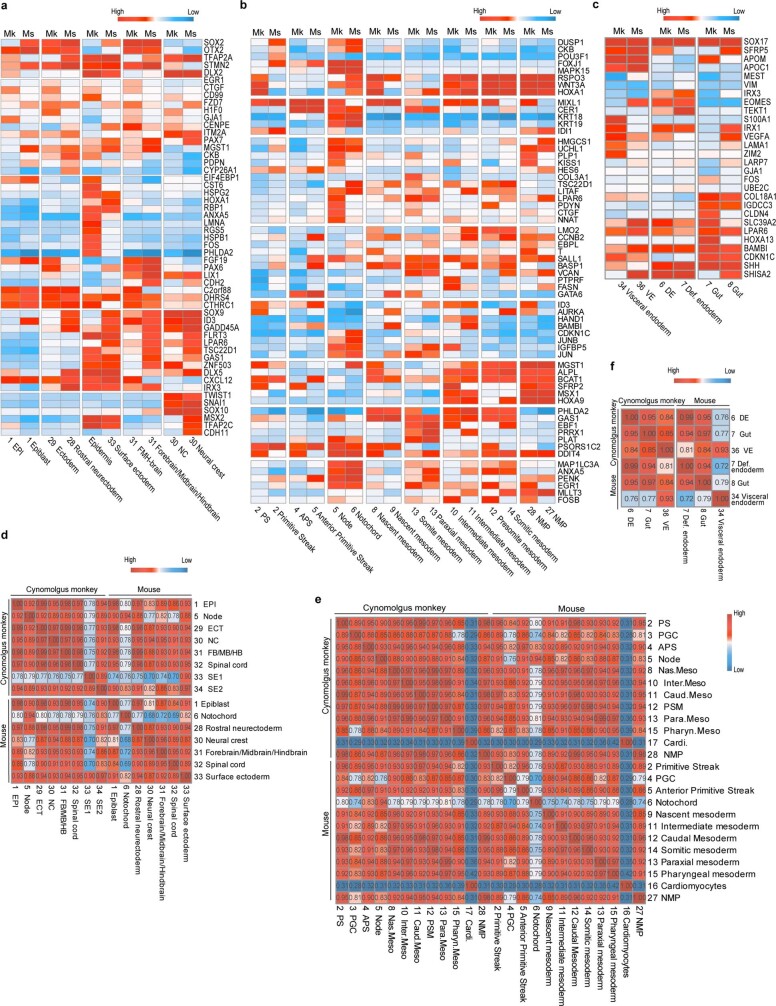
The transcriptomic comparison of three germ layers between monkey and mouse. **a**–**c**, Heatmaps showing the DEGs in indicated monkey (Mk) and mouse (Ms) cell types related with ectoderm (**a**), mesoderm (**b**) and endoderm (**c**) development. Color bars, normalized expression level of different genes from high (red) to low (blue) in indicated cell types. **d**–**f**, Heatmaps of the correlation coefficients among the indicated cells including those reported by mouse dataset based on the cynomolgus monkey ontogenic gene levels, performed by Pearson Correlation Coefficient. Gene expression values of the integrated dataset were calculated by “cor()” in R, here and after. No other statistical analysis. Colors from blue to red indicate the low to high levels of Pearson Correlation Coefficient between each cell types, here and after.

Although mice and monkeys are animal models widely used for understanding human development, single-cell transcriptome comparisons of the earliest steps in organogenesis among mice, monkeys and humans are lacking. To this end, we first annotated the paralogues and one-to-one orthologues from mice^6^, monkeys and humans^2,3^ (Extended Data Fig. 5a,b and Supplementary Tables 4 and 5). By and large, cross-species conserved expression patterns of orthologues were observed during gastrulation and early organogenesis. Notably, *HES4*, known to specify anterior mesoderm within the organizer and to control the proliferation of neural crest and neural cells^35,36^, was the only paralogue found expressed in humans and monkeys but not in mice (Extended Data Fig. 5c). Next, we integrated our CS8–11 monkey embryos scRNA-seq dataset with single-cell transcriptomes derived from mouse embryos at corresponding developmental stages (Theiler stage (TS) 9–12). The scmap analysis suggested that cells from CS8–9 and CS11 monkey embryos were mostly comparable to analogous cell types of TS9–12 mouse embryos (Extended Data Fig. 5d–g). Uniform manifold approximation and projection (UMAP) showed well-matched major cell types from both species (Fig. 4a). Based on this integrated dataset, we performed cross-species comparisons and identified many conserved and divergent transcriptomic features of EPI, PS, APS, primordial germ cells (PGCs), ectoderm (NE, FB/MB/HB, SE, SC and NC), mesoderm (node, Nas.Meso, Inter.Meso, Para.Meso, PSM, NMP) and endoderm (VE, DE and Gut) between monkeys and mice (Extended Data Fig. 6 and Supplementary Tables 6–11).

**Extended Data Fig. 7:**
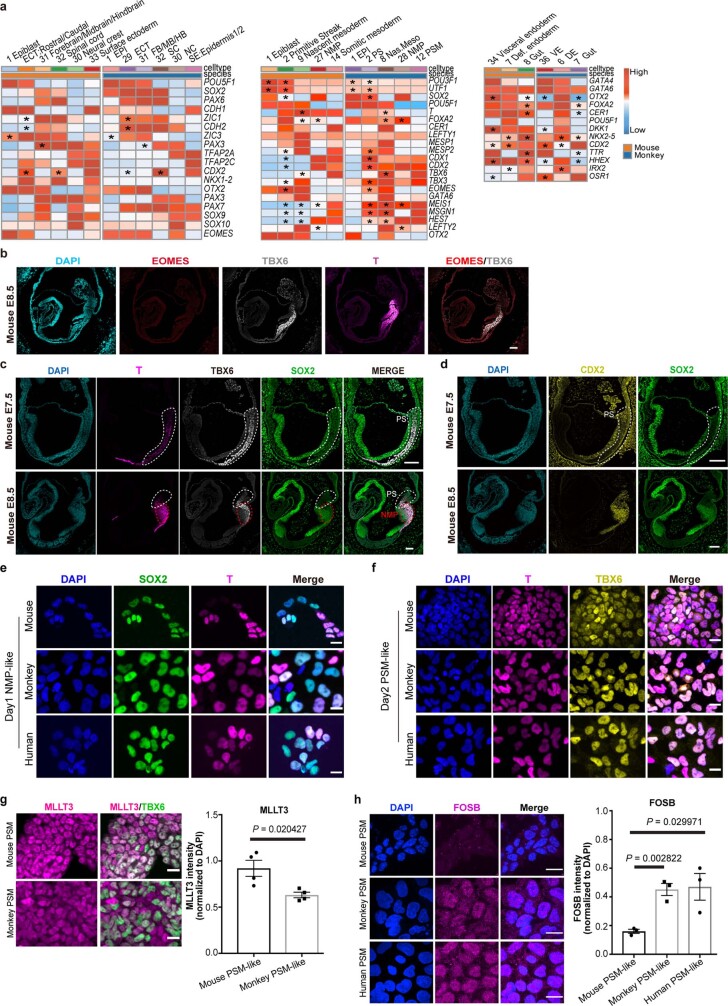
The differences in gene expression pattern between monkey and mouse embryos. **a**, Heatmaps showing the dynamic expression of marker genes in the differentiation of different lineages, including EPI-PS-Nas.Meso-NMP-PSM (left), EPI-ECT-neural (middle), and VE-DE-Gut (right) cells. Black asterisks indicating genes (i.e., *FOXA2, TBX6, EOMES*, and *OTX2*) that show distinct expression patterns between mouse and monkey. Colors from blue to red indicate the low to high expression level of different genes in indicated cell types. **b**, IF results exhibiting the localization of EOMES^Low^/TBX6^+^/T^+^ cells on E8.5 mouse embryos. DAPI, marker for DNA, here and after. Scale bar, 100 µm. **c**, IF results exhibiting the localization of T^+^/TBX6^+^/SOX2^+^ NMP on E7.5-E8.5 mouse embryos. White dashed lines highlight the PS region (SOX2^−^/T^+^/TBX6^+^), here and after. Red dashed lines highlight the potential NMP region (SOX2^+^/T^+^/TBX6^+^). Scale bars, 100 µm. **d**, IF results exhibiting the localization of CDX2^+/Low^/SOX2^+^ cells on E7.5-E8.5 mouse embryos. Scale bars, 100 µm. All the IF experiments (**b**–**d**) were independently repeated in 3 samples. **e**–**f**, IF results showing the lineage-specific markers (SOX2 and T for NMP, T and TBX6 for PSM) of NMP-like and PSM-like cells differentiated from pluripotent stem cells. Scale bars, 20 µm. Experiments were independently repeated 3 times. **g**, (left) IF validation of *MLLT3* captured by scRNA-seq in mouse and monkey PSM-like cells. Scale bars, 20 μm. (right) Bar graph indicating the quantification of MLLT3 immunofluorescent intensity, which is normalized to DAPI. Data are shown as mean ± s.e.m. (n = 4, biological replicates, experiments were independently repeated twice with similar results). Mouse PSM versus monkey PSM, the two-tailed *P* value = 0.020427. **h**, (left) IF validation of FOSB captured by scRNA-seq in mouse and monkey PSM-like cells. Scale bars, 20 µm. (right) Quantification of FOSB immunofluorescent intensity, which is normalized to DAPI. Data are shown as mean ± s.e.m. (n = 3, biological replicates, experiments were independently repeated twice with similar results). The mouse PSM versus monkey PSM, two-tailed *P* value = 0.002822; the mouse PSM versus human PSM, two-tailed *P* value = 0.029971. Source data

To examine differences in cellular developmental dynamics between mouse and monkey embryos, we studied the expression patterns of selected marker genes during different lineage transitions, including EPI→PS→Nas.Meso→NMP→PSM, VE→DE→Gut and EPI→ECT→FB/MB/HB (Extended Data Fig. 7a). Heatmap and IF analyses showed that genes such as T, EOMES and TBX6 exhibited distinct expression patterns between mouse and monkey PS, Nas.Meso, NMP and ectoderm cells (Figs. 2d and 4b and Extended Data Fig. 7a–d). Interestingly, in contrast to mice, many downstream genes of the Hippo signalling pathway were upregulated in monkey NMP and PSM cells (Fig. 4c). To validate and better understand species-specific Hippo signalling activities during PSM differentiation, we took advantage of pluripotent stem cell (PSC)-based in vitro models^37,38^ and recapitulated NMP and PSM differentiation from mouse, monkey and human PSCs (Extended Data Fig. 7e,f). Based on these in vitro models, we confirmed the different expression levels of MLLT3 and FOSB in mouse and monkey PSM-like cells by IF (Extended Data Fig. 7g,h). Consistent with transcriptomic analysis, we found distinct YAP1 localization between mouse (cytoplasm) and monkey/human (nuclear) PSM-like cells, suggesting lower activities of Hippo kinases in monkey/human PSM (Fig. 4d). In agreement, inhibition of the Hippo pathway by lysophosphatidic acid (LPA) severely impaired mouse but not monkey or human PSM differentiation (Fig. 4e, f).

**Extended Data Fig. 8:**
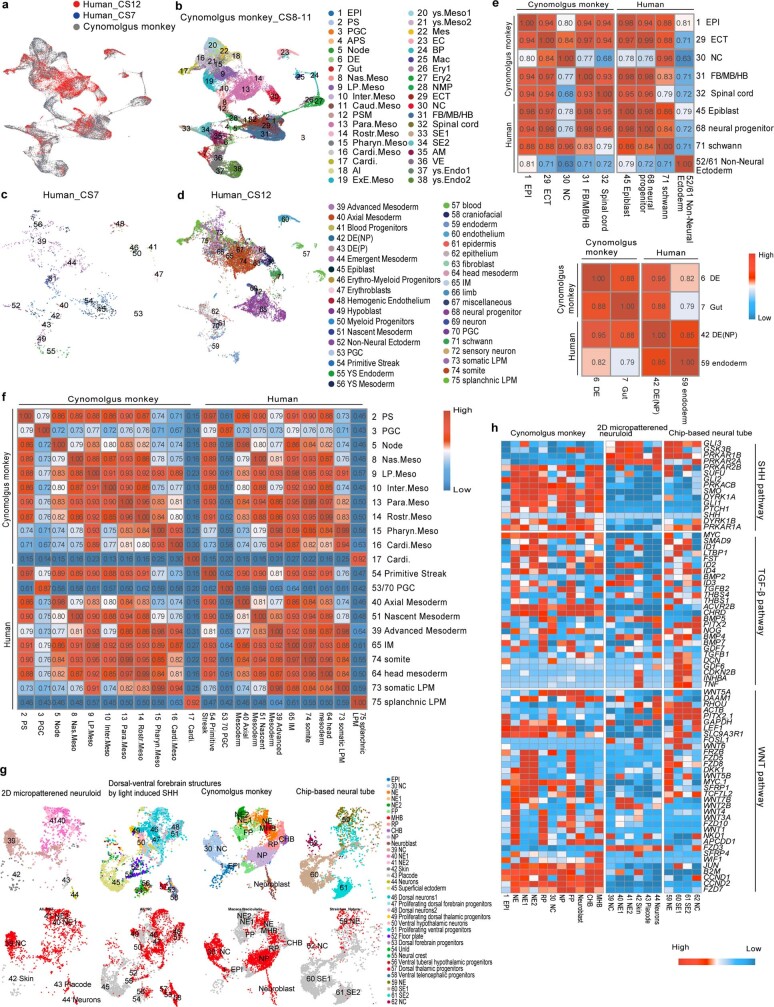
The transcriptomic comparison between monkey, human embryos, and human neuruloids. **a**, UMAP showing the integrated single-cell transcriptome datasets of our monkey CS8–11 embryos and human CS7 and CS12 embryos. **b**, UMAP indicating the 38 cell types from monkey embryonic cells. **c**, UMAP showing the 18 cell types of human CS7 embryo^2^. **d**, UMAP showing the 19 major cell types of human CS12 embryo^3^. LPM, lateral plate mesoderm; IM, intermediate mesoderm. **e**–**f**, Heatmaps of the correlation coefficients among the indicated cells including those reported by human dataset based on the cynomolgus monkey ontogenic gene levels, performed by Pearson Correlation Coefficient. No other statistical analysis. **g**, Comparison of CS11 monkey neural cells and cells of different human neuruloids with dorsal-ventral patterning^47–49^. The top row showing the annotated cell types from different datasets, and the bottom row highlighting the comparable cell types. **h**, Heatmap showing the expression of key genes of the SHH, TGF-β, and WNT signalling pathways enriched in monkey neural cells and human neuruloid cells. Colors from blue to red indicate the low to high expression level of different genes in indicated cell types.

To gain insight into perigastrulation development in humans, several stem cell embryo models have recently been developed^39–41^. To date, the fidelity of most models has yet to be evaluated due to limited reference datasets of human embryos. To determine whether our CS8–11 monkey embryos dataset could serve as a surrogate reference, we combined it with datasets from a CS7 (ref. ^2^) and a CS12 (ref. ^3^) human embryos. Integrated analysis and annotation of cell types confirmed the high conservation of both humans and monkeys during embryonic development (Extended Data Fig. 8a–f).

**Extended Data Fig. 9:**
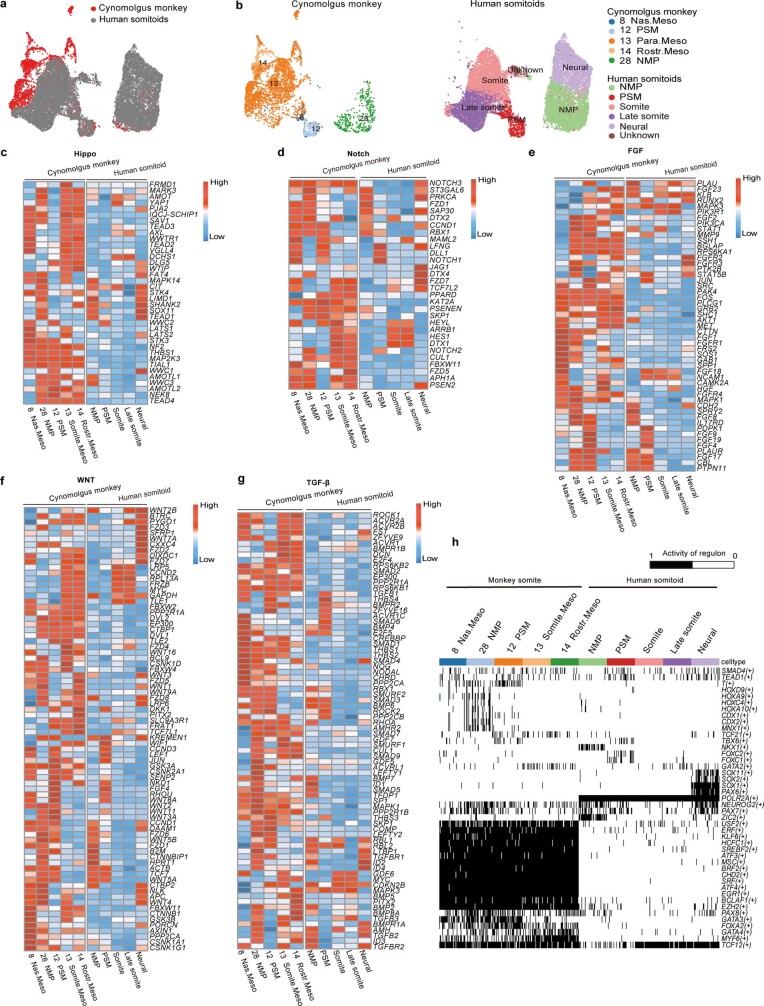
The transcriptomic comparison between monkey segmentation-related cells and human somitoid cells. **a**,**b**, UMAPs showing the comparison of monkey Para.Meso-related cells and human somitoid cells^50^. **c**–**g**, Heatmaps showing the expression of key genes of the Hippo, Notch, TGF-β, WNT, and FGF signalling pathways enriched in monkey Para.Meso-related cells and human somitoid cells. Colors from blue to red indicate the low to high expression level of different genes in indicated cell types. **h**, Heatmap showing the expression of TFs enriched in monkey somite-related cells and human somitoid cells. Color bar, activity of regulons, indicating the expression (black) or non-expression (white) of different TFs.

**Extended Data Fig. 10:**
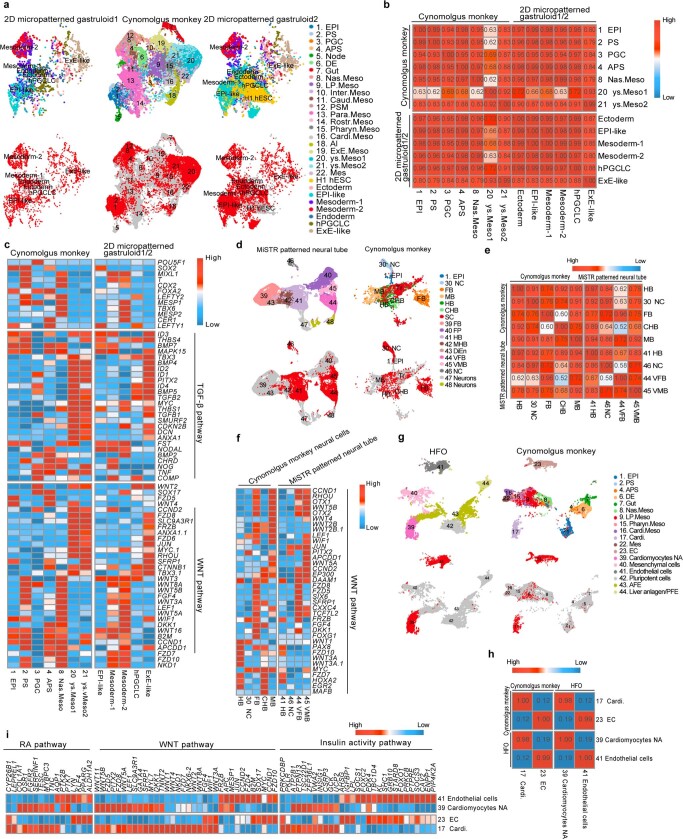
The transcriptomic comparison between monkey embryonic cells and human embryoid (gastruloids, neuruloids with dorsal-ventral patterning, and heart-forming organoid) cells. **a**, UMAPs showing the integrated single-cell transcriptome datasets with human gastruloids^42,43^ projected on monkey map. The top row showing the annotated cell types from different datasets, and the bottom row highlighting the comparable cell types, here and after. H1 hESC, human embryonic stem cell H1; hPGCLC, human primordial germ cell like cells; ExE-like, extra-embryonic mesenchymal-like cells. **b**, Heatmap of the correlation coefficients among the indicated cells including those reported by human dataset based on the cynomolgus monkey ontogenic gene levels, performed by Pearson Correlation Coefficient. No other statistical analysis. **c**, Heatmap showing the expression of specific marker genes in each annotated cell types and key genes of the TGF-β and WNT signalling pathways enriched in monkey embryonic cells and human gastruloid cells. Color bar, the normalized expression of different genes in indicated cell types, here and after. **d**, UMAPs showing comparison of CS11 monkey neural cells and MiSTR^46^ patterned human neuruloid cells. Cells of human neuruloid are compared with cells of different brain regions in monkey embryos. DiEn, diencephalon; VFB, visceral forebrain; VMB, visceral midbrain. **e**, Heatmap of the correlation coefficients among the indicated cells including those reported by human dataset based on the cynomolgus monkey gene levels, performed by Pearson Correlation Coefficient. No other statistical analysis. **f**, Heatmap showing the expression of key genes of the WNT signalling pathway enriched in monkey neural cells and human neuruloid cells. **g**, UMAPs showing comparison of monkey cardiogenesis-related cells and human heart-forming organoid (HFO)^45^. AFE, anterior foregut endoderm. **h**, Heatmap of the correlation coefficients among the indicated cells including those reported by human dataset based on the cynomolgus monkey ontogenic gene levels. **i**, Heatmap showing the expression of key genes of the RA (retinoic acid), WNT and Insulin activity signalling pathways, respectively enriched in monkey cardiogenesis-related cells and human heart-forming organoid cells.

Next, we used the monkey reference to evaluate several human stem cell embryo models. By performing integrated analyses, we found that the main cell types from several gastruloids^42,43^, heart-forming organoids (HFOs)^44,45^, neuruloids (for example, the MiSTR patterned neural tube that mimics the A–P axis^46^ and the two-dimensional micropatterned, light-induced or chip-based neural tube that mimics the D–V axis^47–49^) and somitoids^50^ generally overlapped with the CS8–11 monkey embryos reference (Extended Data Figs. 8g,h, 9 and 10 and Supplementary Tables 12 and 13), although notable differences were observed. Using neuruloids and somitoids as proofs of concept, we performed further in-depth comparative analyses of signalling pathways and TFs and found that expression patterns of TGF-β family members *BMP4*, *BMP5* and *BMP7* and WNT ligands in two neural tube models were, by and large, similar to the monkey reference (Extended Data Fig. 8h). However, neither model recapitulated SHH signalling features observed in monkey embryos, suggesting a lack of D–V patterning (Extended Data Fig. 8h). The major cell types during monkey somitogenesis, including NMP, PSM and Para.Meso, were recapitulated in human somitoids (Extended Data Fig. 9a,b). Interestingly, many genes related to Hippo, WNT, FGF, TGF-β and Notch signalling pathways were found upregulated in monkey embryos when compared with human somitoids (Extended Data Fig. 9c–g). Besides, we identified TFs specifically expressed in monkeys (for example, *EGR1*, *ATF4*, *SRF*, *CHD2*, etc.) and in humans (*POLR2A*) (Extended Data Fig. 9h).

Collectively, these results uncovered conserved and divergent features of embryonic development between monkeys and mice. Moreover, using the CS8–11 monkey embryos dataset as a reference we evaluated several human stem cell embryo models.

## Discussion

In this study, through comprehensive scRNA-seq analyses of 56,636 cells, we identified 38 major cell clusters and unveiled the developmental landscapes of all three primary germ layers present during gastrulation and early organogenesis in primates. We then compared the single-cell transcriptomes of CS8–11 monkey embryos with mouse embryos at comparable developmental stages and gained insights into conserved and divergent transcriptomic features across species. In addition, we demonstrated the utility of the CS8–11 monkey embryos dataset as an in vivo reference for authentication of human stem cell embryo models.

The scarcity of CS8–11 human embryos for research, during which primordial organs and the body plan are established, has led to reliance on animal and stem cell embryo models to study this enigmatic period of human embryo development. NHPs, due to their evolutionary resemblance to humans, provide the closest proxy to understanding human embryogenesis. Our comprehensive single-cell transcriptome atlas of a NHP species through CS8 to CS11 not only bridges the knowledge gap in primate embryogenesis but also expands the collection of embryo datasets for comparative developmental biology and benchmarking of embryoid and organoid models.

## Methods

### Ethical statement

This study was conducted in accordance with the Principles for the Ethical Treatment of Non-Human Primates issued by the Institute of Zoology, Chinese Academy of Sciences (IOZ, CAS), and was approved in advance by the Institutional Animal Care and Use Committee of IOZ, CAS (no. IOZ-EU-20191113 for all monkey experiments, no. IOZ-IACUC-2021-037 for all mouse experiments). Both followed relevant guidelines and regulations. hESC experiments in this study were performed at the UT Southwestern Medical Center and followed the International Society for Stem Cell Research guidelines for Stem Cell Research and Clinical Translation, 2021 (https://www.isscr.org/policy/guidelines-for-stem-cell-research-and-clinical-translation). hESC work was reviewed and approved by the UT Southwestern Stem Cell Oversight Committee.

### Experiment models and biological sample preparation

#### Collection of embryonic samples

All *Macaca fascicularis* were of Southeast Asian origin. The animals were maintained at around 25 °C on a 12/12-h light/dark schedule and raised at the Xieerxin Biology Resource with the accreditation of the laboratory animal care facility in Beijing. All animals were given a commercial diet twice per day with tap water ad libitum and were fed vegetables and fruits once daily under careful veterinary supervision. Before the experiment, none of the animals had a clinical or experimental history that would affect physiological ageing or increase susceptibility to diseases.

Oocyte collection, intracytoplasmic sperm injection, pre-implantation embryo culture and transfer of pre-implantation embryos to foster mothers were performed as described by Yamasaki et al.^51^. Briefly, female cynomolgus monkeys around 6–8 years of age were chosen for oocyte collection by superovulation with follicle-stimulating hormone, and an implantable and programmable microfusion device was implanted subcutaneously under ultrasound detection. The day when the collected ova were artificially fertilized by sperm injection was designated as embryonic day 0 (E0). When the embryos developed with blastocoel cavities around E6–7, five or six high-quality embryos were selected and transferred to appropriate recipient female cynomolgus monkeys. The implanted embryos were further monitored by ultrasound scanning from E14 to identify successful pregnancies. Ketamine hydrochloride (0.1–0.2 ml kg^–1^) was administered by intramuscular injection for the anaesthesia of pregnant monkeys. The implanted uterus was surgically removed at different developmental stages asexperimentally designed, from which embryonic tissues could be obtained. The sample size of the study was determined based on the availability of highly regulated primate embryo samples. In compliance with the 3R guidelines we reduced the number of animals used to a minimum, which allowed us to obtain a high-coverage transcriptome for each cell type and confidently perform downstream analyses.

C57BL/6 mice were housed under a 12/12-h light/dark cycle at around 25 °C. Natural mating was established between males and 6–8-week-old females, with 12:00 on the day of vaginal plug insertion considered to be E0.5. Postimplantation embryos were dissected from uteri at E7.5–8.5 for the experiments described below.

#### Isolation of embryonic cells

Monkey embryonic tissues were transferred to DMEM/F12 (DF12) medium (Gibco, no. 21331020) containing 5% Penicillin-Streptomycin (Gibco) and stored at 4 °C for a short period. After washing in PBS (Gibco), tissues were digested with 0.125% TrypLE (Gibco) and 0.025% DNase (Gibco) in DF12 at 37 °C with stirring for 10 min. The disaggregated cell suspension was passed through 40-μm sterilesieve mesh and washed thoroughly with DF12 containing 10% fetal bovine serum (Invitrogen). Sieved cells were precipitated and collected by centrifugation at 300 *g* for 5 min. Precipitated cells were resuspended with 5 ml of red blood cell lysis buffer for 3 min and then diluted with an additional 25 ml of DF12 medium. After removal of red blood cells, cells were recentrifuged and transferred to short-term storage at 4 °C.

#### Preparation of scRNA-seq library and sequencing

Single-cell libraries were constructed using Single Cell 3 Library & Gel Bead Kit v.3 according to the manufacturer’s protocol (10X Genomics)^52^. In short, cell counts were assessed busing a haemocytometer (Luna-FL, Logos Biosystems) with cell concentration adjusted to 1,000 μl^–1^. About 16,000 cells were added to each channel of a 10X loading chip and then around 8,000 were captured. Captured cells were lysed, and the isolated RNA was barcoded through reverse transcription in individual gel bead in the emulsion. cDNA was then amplified to construct the library and the qualities of cDNA and cDNA libraries were assessed using Agilent 2100. Finally, the libraries were sequenced on an Illumina Hiseq X Ten platform (Annoroad Gene Technology).

### Single-cell transcriptomic analysis

#### scRNA-seq data preprocessing

Raw fastq files were processed using Cell Ranger 3.1.0 software with default mapping arguments^52^. Reads were mapped to the *Macaca fascicularis* 5.0 genome. Next, the CellRanger ‘aggr’ command was used to normalize the sequencing depth of different samples, with mean reads per cell above 30,220 post normalization.

#### Filtering of cells, integration, dimensionality reduction and clustering

The filtered expression matrix with cell barcodes and gene names was loaded with the ‘Read10X’ function of the Seurat (v.4.0.0) R package^53^. First, single cells with the number of detected genes (nFeature_RNA) above 500 and detected transcripts (nCount_RNA) above 1,000 were retained to exclude apoptotic or dead cells. Next, doublet or multiplet cells were determined with Scrublet, according to the recommended multiplet rate reference table from 10X Genomics^54^. Next, Seurat objects of different samples (seven samples, Supplementary Table 1) were created independently, with the expression matrix and metadata containing cell barcodes, and cell multiplet information inferred by Scrublet, followed by merging of these Seurat objects. For monkey genes poorly annotated, gene names annotated by *Macaca fascicularis* 5.0 were further converted to those of human-based genes on the published annotation information to better interpret the data^4^. After exclusion of doublet or multiplet cells, 56,636 embryonic cells remained. Next, we used the dataset integration function of Seurat^53^ to exclude individual heterogeneities between different monkeys. In brief, after normalization of the Seurat object we selected highlyvariably expressed genes by the ‘mean.var.plot’ method at the FindVariableFeatures step, with 2,117 genes found to have highly variable features. These feature genes of anchor and default 30 dimensions of canonical correlation analysis were used for FindIntegrationAnchors, IntegrateData, RunPCA and so on. A tree number of 50 was set as as default when finding integration anchors. Subsequently, the Seurat pipeline was used for dimensionality reduction (UMAP) and unsupervised clustering. In most cases we used the default settings of Seurat during dimensionality reduction and unsupervised clustering. To construct the UMAP plot we selected the number of dimensions mainly according to the ‘ElbowPlot’ function. For UMAP of 56,636 embryo cells we used the first 16 principal component analysis dimensions at the RunUMAP procedure; the seed used was 42, minimum distance was 0.3 and n.neighbors was 30 as the default setting of Seurat v.4, except that ‘umap.method’ was ‘umap-learn’ and the metric was ‘correlation’. For clustering of the 56,636 embryo cells the ‘k.parameter’ of 20 and ‘n.trees parameter’ of 50 were the default settings during the neighbour-finding process; the number of dimensions used for neighbour finding was 16, as also used for UMAP construction. A resolution of 0.9 was used at the ‘FindClusters’ step, as shown in Fig. 1b, different types of single cells grouped well.

#### Differentially expressed gene (DEG) and Gene Ontology (GO) analyses

We computed the DEGs of each cell cluster with RNA assay using the FindAllMarkers function of the Seurat package^53^. Heatmaps were plotted based on the top ten highly expressed genes (according to adjusted *P* values and fold change) of each cell cluster. The DEGs of each cell cluster from mouse and monkey were used for GO enrichment and analysed by the clusterProfiler R package^55^. GO terms were enriched by the ‘compareCluster’ function, and ‘ont=BP’ was set.

#### Pseudotime analysis

The ‘monocle3’ R package^56^ was used to calculate the developmental pseudotime of single cells. The Seurat object was converted to a monocle3 object by the ‘as.cell_data_set’ command of the SeuratWrappers R package^53^. The developmental trajectory was then constructed with the ‘learn_graph’ function of the monocle3 R package. After setting the developmental starting point, the ‘order cells’ command was used to analyse developmental pseudotimes. Finally, pseudotime trajectory was visualized with the ‘plot_cells’ function.

#### RNA velocity analysis

Read annotations for sequenced samples were performed using the ‘velocity run 10X’ command-line tool with BAM, genome annotation and repeat annotation files^8^. BAM files were generated by the default parameters of Cell Ranger software (10X Genomics)^52^. *Macaca fascicularis* 5.0 genome annotations were used to count molecules while separating them into three categories: spliced, unspliced or ambiguous. Repeat annotation files were downloaded from the UCSC genome browser. We then used the UMAP embedding matrix computed by the Seurat pipeline to construct the velocity map with the scVelo python package^8^. Briefly, the loom file containing three categories of count value was loaded to the R environment by the ‘ReadVelocity’ function of the SeuratWrappers package when the Seurat pipeline was completed. These data were added to the Seurat object, after which the Seurat object was converted to the ‘h5ad file’ with the SeuratDisk R package^53^ and the ‘h5ad’ file was loaded by the ‘scv.read’ function of the scVelo python package^57^. After the h5ad file was further filtered and normalized, ‘pp.moments’, ‘tl.velocity’ and ‘tl.velocity_graph’ commands were executed to compute RNA velocities. Finally, using the function ‘pl.velocity_embedding_stream’, RNA velocity vectors were projected onto to the UMAP produced by the Seurat pipeline.

To address concerns about 10X sequencing depth, PS-mesoderm lineage cells (Extended Data Fig. 2b) were divided into 282 microclusters by the Seurat unsupervised clustering method (resolution, 50) based on transcriptomic similarities. Spliced and unspliced transcripts of each microcluster were further merged (the sum of corresponding transcript count values in all cells of each microcluster was computed separately), then each microcluster was treated as a ‘pseudocell’. After microclustering, the new Seurat object was recreated with the merged nCount data and pseudocells were annotated according to the maximum cell population of each microcluster. The total number of detected genes and UMI per cell were increased (nCount (UMI), 1 × 10^5^ unspliced and 4 × 10^5^ spliced; nFeature (genes), 10,000 unspliced and 12,000 spliced), which was helpful in regard to compensating for the depth shortage of 3’ sequencing. After the Seurat pipeline, UMAP coordinates were substituted with mean UMAP values of cells in each original microcluster. RNA velocity vectors were then computed with the scVelo python package^8^. The validation of velocity on endoderm lineage based on microclustering was performed using a similar method. In addition, ‘Velocity_True’and ‘Velocity_False’ genes were exported from the ‘velocity_genes’ of the scVelo object, and ‘Conflict’ genes were computed based on the methods of Barile et al.^58^.

#### Pseudotime trajectory analysis of Gut

The Seurat object with scale data of Gut was converted to the h5ad file by the SeuratDisk (v.0.0.0.9013) R package^59^, and the h5ad file was then loaded to the python environment by the ‘sc.read’ function of the Scanpy (v.1.8.2) python package^60^. Thereafter, principal components were recomputed with the ‘tl.pca’ function of Scanpy. The Force-directed graph was constructed with the 14 nearest neighbours with default principal components of the scale data (using the Scanpy ‘tl.draw_graph’ function), and the layout was generated with the ForceAtlas2 algorithm^61^. Graph abstraction was computed with the ‘tl.paga’ function of Scanpy v.1.8.2. The PAGA plot was drawn with the ‘pl.paga_compare’ function for improved correlation of cell clusters to the Force-directed graph. The threshold for connection of clusters was set to 0.15, node size scale to 3 and edge width scale to 0.8. Diffusion pseudotime^62^ was computed using the ‘tl.dpt’ function of Scanpy, with cluster 1 set as the starting point.

#### TF analysis

The pySCENIC analysis in Docker was carried out following three steps^63^. The gene expression matrix was converted to loom file by the ‘loompy’ in python, then the ‘pyscenic grn’, ‘pyscience ctx’, and ‘pyscience aucell’ were used to infer the gene regulatory network, regulon prediction and cellular enrichment (area under the curve, AUC) processes with the corresponding cells. After gene regulatory network was produced by ‘pyscenic grn’, the regulon specificity scores were computed based on the cell clusters identified by Seurat, and we chose top regulons for each cell cluster following ‘pyscience ctx’. The AUC matrix was used to score regulon activity of each cell. The AUCell scores identified important regulons in cells by “pyscience aucell”. The result was a binary regulon activity matrix (binarized activity matrix) that determined in which cells Regulon is ‘on’. The SCENIC AUC heatmap was plotted with binarized activity regulons of each cell cluster by the ‘pheatmap’ R package with the annotation information in the Seurat object.

#### Cell–cell communication analysis

Cell annotation information and raw count expression matrix were exported from the Seurat file with suggested scripts using the CellPhoneDB protocol^64,65^. Cell annotation information and count expression matrix were then used as input for CellPhoneDB statistical analysis with default settings, and this step together with the following plotting step was executed at the Linux command-line interface supported by the protocol. The database of receptor–ligand interactions was generated for human proteins, and the genes of the monkey have been transferred to human genes at the maximum extent to minimize differences in receptor–ligand interactions that might vary between monkeys and humans. Finally, we showed some notable interactions between relevant cell types with the dot-plot function of CellPhoneDB.

#### Comparison of single-cell transcriptomic dataset among mouse, human and monkey

To project monkey single-cell data onto the mouse UMAP, the mouse single-cell reference dataset was first prepared (Fig. 4a). scRNA-seq data of early mouse embryogenesis^6^ were obtained from EMBL-EBI ArrayExpress under experiment code no. E-MTAB-6967. The count expression matrix and cell annotation files supplied were used to create the Seurat object with the Seurat (v.4.0.0) R package^59^. Using the method of Blanca Pijuan-Sala et al.^6^, 116,312 single-cell transcriptomes remained. The mouse Seurat object (reference dataset) was created with 13,805 monkey/mouse shared genes. Following the RunUMAP procedure (return.model, TRUE), UMAP cell-embedding values were replaced by those supplied in the cell annotation file of no. E-MTAB-6967. Cell clusters in the UMAP plot, as shown in Fig. 4a (right), were also annotated according to the annotation files supplied. The monkey Seurat object (query dataset) was also created, with 13,805 monkey/mouse shared genes. The anchors between mouse and monkey data were found with the FindTransferAnchors function (reference.reduction, ‘pca’; dims, 1:50; k.filter, NA), and the function MapQuery (reference.reduction, ‘pca’; reduction.model, ‘umap’) was used to project monkey embryo single-cell data onto the mouse embryo single-cell data-based UMAP structure. The cell clusters shown in Fig. 1b are shown in the projected UMAP plot in Fig. 4a (left).

To do integration analysis for monkey and human single-cell data, the human CS7 embryo^2^ and various embryoid datasets including gastruloids^42,43^, HFOs^45^, neuruloids^46–49^ and somitoids^50^ were prepared (Extended Data Figs. 8a–d,g, 9a,b and 10a,d,g). We used the ‘biomaRt’ package to convert genes from cynomolgus monkeys and mice to human homologous genes^53^. Seurat lists were split by samples or species, and each list was normalized using ‘NormalizeData’ function. Next, 2,000 genes were selected as anchor features. Using the R package ‘Seurat’ with the functions ‘FindIntegrationAnchors’ and ‘IntegrateData’, based on canonical correlation analysis and mutual nearest-neighbours algorithms, we acquired the integration Seurat objects of cynomolgus monkeys with mice then set the default assay as ‘integrated’. UMAPs were calculated using the function of ‘RunUMAP’ with dimensions set as 30. The same methods were performed for integration between natural monkey and human embryos^2^ (or human embryoids).

#### Developmental staging of monkey and mouse embryos

We selected EPI, rostral neuroectoderm, SE, forebrain/midbrain/hindbrain and NC from the E6.5–8.5 mouse embryo dataset^6^ and compared them with their counterpart cells in CS8–11 cynomolgus monkey embryos using the ‘scmap’ R package^4,66^. Furthermore, we selected 1,000 genes by setting ‘n_featurre=1000’ in the function ‘selectFeatures’ with the parameter threshold set to 0 in ‘scmapCluster’. Default parameters were used for all other steps. We performed the same strategy for the developmental stage comparison of mesoderm and endoderm between cynomolgus monkeys and mice.

#### Comparison of cellular signalling pathways across species

We downloaded gene information for the WNT, FGF, TGF-β, SHH, Hippoand Notch signalling pathways from the MSigDB database^67^. Gene expression of various cell types was detected in cynomolgus monkey, human and mouse embryos and in human stem cell embryo models. Gene expression was scaled from −1 to 1 from the integrated data, and average expression level was measured by cell type using the ‘AverageExpression’ function in the Seurat R package.

### IF analysis on paraffin-fixed embryo sections

Embryonic samples (monkey E22 and mouse E7.5–8.5 embryos) were immediately fixed in 4% paraformaldehyde overnight at 4 °C and subsequently embedded in paraffin. Sections (5 µm) on slides were dewaxed and rehydrated with xylene and ethanol gradients. Slides were immersed in 0.01 mol l^–1^ citric acid buffer solution (C_6_H_8_O_7_.H_2_O:C_6_H_5_Na_3_O_7_.2H_2_O, 1:9, pH 6.0) and heated in a microwave oven at 92–98 °C for 15 min for antigen retrieval. After cooling to room temperature, the slides were washed three times with 1× PBS (5 min each), incubated with 1% Triton X-100 for 30 min and blocked with 2% bovine serum albumin (BSA) for 30 min at room temperature. Next, the slides were incubated with primary antibodies (Supplementary Table 14), diluted with blocking solution overnight at 4 °C and washed three times with PBST (1× PBS with 0.05% Tween-20, 5 min each). The slides were then incubated with secondary antibodies diluted with blocking solution and 1 mg ml^–1^ DAPI (Invitrogen, no. D3571) for 1 h. Finally, after washing three times with PBST (5 min each) the slides were mounted with anti-fade mounting medium (Gibco). IF images were captured by laser-scanning confocal microscope LSM 880 (Carl Zeiss) and processed with Imaris 9.0.2 software (Bitplane) and Zen 7.0 (Carl Zeiss).

### Validation by stem cell models

#### Pluripotent stem cell culture

Human embryonic stem cell line H9 (WA09) was obtained from WiCell and authenticated by short tandemrepeat profiling. Mouse epiblast stem cells (mEpiSCs) and rhesus macaque ES cells were generated and identified as described in a previous study^68,69^. Mycoplasma testing for cell lines was negative. hESCs were maintained in mTeSR Plus medium (STEMCELL Technologies) in a 0.5% Matrigel (BD Biosciences)-coated culture dish at 37 °C and in 5% CO_2_. hESCs were dissociated with accutase (STEMCELL Technologies) and split in a 1:10 ratio. A single-cell suspension was seeded into a Matrigel-coated dish in mTeSR Plus medium containing 10 µM ROCK inhibitor (no. Y27632, Sigma-Aldrich). mEpiSCs were maintained on mouse embryonic fibroblast (MEF) feeder cells in a gelatin-coated dish, in NBFR medium containing DF12 and Neurobasal medium (Invitrogen) mixed in a 1:1 ratio, 0.5× N2 supplement (Invitrogen), 0.5× B27 supplement (Invitrogen), 2 mM GlutaMax (Gibco), 1× nonessential amino acids (NEAA, Gibco), 0.1 mM 2-mercaptoethanol (Sigma-Aldrich), 20 ng ml^–1^ FGF2 and 2.5 μM IWR1. mEpiSCs were dissociated with TrypLE (ThermoFisher) and split in a 1:30 ratio. Rhesus macaque ES cells were maintained on mouse embryonic fibroblast feeder cells in a gelatin-coated dish, in NBFR medium supplemented with 5 mg ml^–1^ BSA (MP Biomedicals). Cells were dissociated with TrypLE and split in a 1:10 ratio. A single-cell suspension was seeded in NBFR (5 mg ml^–1^ BSA) medium containing 10 µM ROCK inhibitor.

#### In vitro differentiation of PSM-like cells

Presomitic mesoderm differentiation was carried out as described in a previous study^37^. On the day of differentiation (day 0), 100,000–150,000 cells (10,416–15,625 cells cm^–2^) were seeded in a Matrigel-coated, 35 mm dish in pluripotency maintenance medium. Cells were maintained in incubator at 37 °C for about 2 h before changing to differentiation medium; differentiation medium contains DF12 with 1× N2 supplement and Neurobasal medium (Invitrogen) with 1× B27 supplement in a 1:1 ratio. The medium was also supplemented with 2 mM Glutamax, 0.1 mM nonessential amino acids, 1 mM sodium pyruvate (Gibco), Penicillin-Streptomycin (Gibco), 10 µM CHIR99021 (Selleckchem) and 0.5 μM LDN193189 (Selleckchem). Differentiation medium was changed daily. The same protocol was used for differentiation of human, monkey and mouse PSM-like cells. For Hippo inhibition experiments, cells were treated with 0.5 µM 1-Oleoyl LPA (OCRIS, no. 3854) on day 1.

#### IF staining and microscopy

For IF staining, 10,416–15,625 cells cm^–2^ were initially seeded on Matrigel-coated µ-Slide eight-well chambered coverslips (ibidi, for high-end microscopy). The cells were fixed at the indicated time points (days) in 4% paraformaldehyde for 15 min at room temperature. Fixed cells were washed twice with PBS before permeabilization and blocking with 3% donkey serum in PBST (1× PBS with 0.1% Triton X-100) for 1 h at room temperature. Samples were incubated with primary antibody (Supplementary Table 14) and diluted in blocking buffer at room temperature for 2 h or 4 °C overnight followed by 30 min of PBST wash, repeated twice. Secondary antibodies (ThermoFisher) were diluted in blocking buffer in a 1:500 ratio. DAPI staining was performed, together with secondary antibodies, at room temperature for 1 h followed by three PBST washes. Samples were then soaked in PBS before imaging. Fluorescence imaging was performed on either (1) a Nikon CSU-W1 SoRa spinning-disk confocal microscope with objectives ×20/0.45 numerical aperture (NA), WD 8.9–6.9, air, ×40/0.6 NA, WD 3.6–2.85, air and ×100/1.45 NA, oil or (2) a Zeiss LSM 800 laser-scanning confocal microscope with a ×40/1.3 NA oil objective.

#### Imaging and statistical analysis

Statistical analyses were repeated at least twice, with consistent results. In the figure captions *n* denotes the number of biological replicates in the same experiment. Raw images were first processed in Fiji^70^ to create maximal intensity projection (MIP) and export look-up table of representative images. For MLLT3 and FOSB data shown in Extended Data Fig. 7g,h, nuclear segmentation was performed in Ilastik^71^. MIP images and segmentation masks were processed in MATLAB (R2022a) using custom code, which is available in a public repository. Nuclear localized fluorescence intensity of transcription factors was computed for each cell in a given field, and the value was then normalized to the DAPI intensity of the same cell. Values of all cells were plotted as mean ± s.e.m. For data shown in Fig. 4f, total cell and TBX6-positive cell numbers were calculated with Imaris (v.9.9, Oxford Instruments) using the SPOTS function. Total cell number was calculated by counting the number of nuclei (DAPI). The same parameters for computation of the spots were applied to the DAPI and TBX6 channels. Data were shown as mean ± s.d. *P* values were determined by unpaired *t*-test. GraphPad Prism v.7.0 was used to plot the data shown in Fig. 4f and Extended Data Fig. 7g,h.

### Reporting summary

Further information on research design is available in the Nature Portfolio Reporting Summary linked to this article.

## Online content

Any methods, additional references, Nature Portfolio reporting summaries, source data, extended data, supplementary information, acknowledgements, peer review information; details of author contributions and competing interests; and statements of data and code availability are available at 10.1038/s41586-022-05526-y.

## Supplementary information

Reporting Summary

Supplementary Table 1 Sample list of cynomolgus monkey embryonic tissues at CS8-CS11. This table summarizes the basic information about the embryonic samples, including the sample name, gender, developmental stages, number of collected cells based on the 10X Genomic Chromium platform, and the number of filtered cells for the following analysis.

Supplementary Table 2 The cell number of different subtypes from 7 samples. Full names and abbreviations of the 38 clusters we annotated and their cell numbers from each embryonic sample.

Supplementary Table 3 Marker genes of different subtypes. Full names and abbreviations of the 38 clusters we annotated and their specific marker genes.

Supplementary Table 4 Orthologues differentially expressed between human, monkey and mouse. The top 50 differentially expressed genes (DEGs) of orthologues in human, monkey and mouse embryonic cells, performed by Wilcoxon Rank Sum test, two-sided, based on Bonferroni correction.

Supplementary Table 5 Orthologues of transcriptome factors (TFs) among human and monkey embryos. The 148 orthologues of TFs among human and monkey embryos. Zinc finger (ZNF) genes are overrepresented among human and macaque monkey orthologues of TFs. No other test was required for the analysis on the homologous transcription factors of the three species.

Supplementary Table 6 The DEGs between monkey and mouse PS and mesoderm cells. Top 20 DEGs in indicated cynomolgus monkey and mouse PS and different mesoderm cell types, performed by Wilcoxon Rank Sum test, two-sided, based on Bonferroni correction.

Supplementary Table 7 Gene ontology (GO) analysis based on DEGs between monkey and mouse primitive streak and mesoderm cells. Representative GO terms enriched in DEGs upregulated in indicated monkey and mouse PS and mesoderm cell types, performed by Fisher test, one-sided, based on Benjamini-Hochberg correction.

Supplementary Table 8 DEGs between monkey and mouse ectoderm-derived cells. The top 20 DEGs in indicated cynomolgus monkey and mouse ectoderm-derived cell types, performed by Wilcoxon Rank Sum test, two-sided, based on Bonferroni correction.

Supplementary Table 9 GO analysis based on DEGs between monkey and mouse ectoderm-derived cells. Representative GO terms enriched in DEGs upregulated in indicated monkey and mouse ectoderm-derived cell types, performed by Wilcoxon Rank Sum test, two-sided, based on Bonferroni correction.

Supplementary Table 10 DEGs between monkey and mouse endoderm-derived cells. Top 20 DEGs in indicated cynomolgus monkey and mouse endoderm-derived cell types, performed by Wilcoxon Rank Sum test, two-sided, based on Bonferroni correction.

Supplementary Table 11 GO analysis based on DEGs between monkey and mouse endoderm-derived cells. Representative GO terms enriched in DEGs upregulated in indicated monkey and mouse endoderm-derived cell types, performed by Wilcoxon Rank Sum test, two-sided, based on Bonferroni correction.

Supplementary Table 12 Differentially enriched TFs between human 2D micropatterned neuruloid cells and monkey neurulation-related cells. This table summarizes the in-depth analysis of conservation and differentiation in TFs between monkey neurulation-related cells and human micropatterned neuruloid cells.

Supplementary Table 13 Differentially enriched TFs between human somitoid cells and monkey segmentation-related cells. This table summarizes the in-depth analysis of conservation and differentiation in TFs between monkey segmentation-related cells and human somitoid cells.

Supplementary Table 14 Antibodies. Commodity information for the primary and secondary antibodies used in this study.

## Data Availability

The dataset of primate gastrulation and early organogenesis generated in the current study is available in the NCBI Gene Expression Omnibus (GEO) under accession no. GSE193007. The dataset of mouse gastrulation and early organogenesis used as reference is available at ArrayExpress under accession no. E-MTAB-6967. The datasets of human CS7 and CS12 embryos used as reference are available at ArrayExpress under accession no. E-MTAB-9388 and at GEO under accession no. GSE157329. The datasets of human neuruloids are available at GEO under accession nos. GSE118682, GSE173492 and GSE163505. The dataset of human somitoids is available at the ArrayExpress database under accession code E-MTAB-11334. The dataset of human gastruloids is available at GEO under accession nos. GSE144897 and GSE169074. The dataset of heart-forming organoids is available at GEO under accession no. GSE150202. The dataset of MiSTR patterned human neuruloids is available at GEO under accession no. GSE135399. Source data are provided with this paper.

## Source data

Source Data Fig. 4
Source Data Extended Data Fig. 7.

## Code availability

Custom MATLAB code for image processing is available in a public repository (https://github.com/ecamacho90/BiologicalImageProcessing, 10.5281/zenodo.7367663). The other codes are available upon request.
