# Supplementary material for: Primate gastrulation and early organogenesis at single-cell resolution
Source: Nature. 2022 Dec 14;612(7941):732–8. doi: 10.1038/s41586-022-05526-y (PMC9771819; doi:10.1038/s41586-022-05526-y)
Supplement: Supplementary file 1 — Reporting Summary [file 41586_2022_5526_MOESM1_ESM.pdf]

nature portfolio

Corresponding author(s): Hongmei Wang, Jun Wu, Fan Guo, Xiangxiang Jiang

Last updated by author(s): Nov 27, 2022

Reporting Summary

Nature Portfolio wishes to improve the reproducibility of the work that we publish. This form provides structure for consistency and transparency in reporting. For further information on Nature Portfolio policies, see our Editorial Policies and the Editorial Policy Checklist.

Statistics

For all statistical analyses, confirm that the following items are present in the figure legend, table legend, main text, or Methods section.

n/a	Confirmed	
☐	☒	The exact sample size (*n*) for each experimental group/condition, given as a discrete number and unit of measurement
☐	☒	A statement on whether measurements were taken from distinct samples or whether the same sample was measured repeatedly
☐	☒	The statistical test(s) used AND whether they are one- or two-sided<br>*Only common tests should be described solely by name; describe more complex techniques in the Methods section.*
☒	☐	A description of all covariates tested
☐	☒	A description of any assumptions or corrections, such as tests of normality and adjustment for multiple comparisons
☐	☒	A full description of the statistical parameters including central tendency (e.g. means) or other basic estimates (e.g. regression coefficient) AND variation (e.g. standard deviation) or associated estimates of uncertainty (e.g. confidence intervals)
☐	☒	For null hypothesis testing, the test statistic (e.g. *F*, *t*, *r*) with confidence intervals, effect sizes, degrees of freedom and *P* value noted<br>*Give P values as exact values whenever suitable.*
☒	☐	For Bayesian analysis, information on the choice of priors and Markov chain Monte Carlo settings
☒	☐	For hierarchical and complex designs, identification of the appropriate level for tests and full reporting of outcomes
☐	☒	Estimates of effect sizes (e.g. Cohen's *d*, Pearson's *r*), indicating how they were calculated

Our web collection on statistics for biologists contains articles on many of the points above.

Software and code

Policy information about availability of computer code

Data collection	Single-cell libraries were constructed using the Single cell 3’ Library & Gel Bead Kit v3 of 10X Genomics, and were sequenced on an Illumina Hiseq X Ten platform.
Data analysis	We used existing published sequence analysis packages and methods, as detailed in the Methods, including "CellRanger3.1.0", "Seurat (v4.0.0)", Scrublet(0.2.1),"clusterProfiler(v4.0.5)","monocle3 (v1.0.0)", "SeuratDisk (v0.0.0.9013)", "Scanpy(v1.8.2)","SeuratWrappers(0.3.0)", "scVelo(0.2.4)", "pySCENIC(v0.10.0)", "CellPhoneDB(2.1.7)", "biomaRt (v2.46.3)", "scmap(v1.20.0)", Matlab (R2019a), and GraphPad Prism (7.0).<br>Custom MATLAB code for image processing is available in a public repository (https://github.com/ecamacho90/BiologicalImageProcessing, https://doi.org/10.5281/zenodo.7367663). The other codes are available upon request.

For manuscripts utilizing custom algorithms or software that are central to the research but not yet described in published literature, software must be made available to editors and reviewers. We strongly encourage code deposition in a community repository (e.g. GitHub). See the Nature Portfolio guidelines for submitting code & software for further information.

Data

Policy information about availability of data

All manuscripts must include a data availability statement. This statement should provide the following information, where applicable:

- Accession codes, unique identifiers, or web links for publicly available datasets
- A description of any restrictions on data availability
- For clinical datasets or third party data, please ensure that the statement adheres to our policy

Raw data and processed data were uploaded to the NCBI Gene Expression Omnibus (GEO) database with the accession number GSE193007.

Datasets used as references include:
mouse E6.5-E8.6 data:E-MTAB-6967;
human CS7 data: E-MTAB-9388;
human CS12 embryo data: GSE157329;
human gastruloids: GSE144897 and GSE169074;
human MiSRT neuruloid data: GSE135399;
human 2D micropatterned neuruloid: GSE118682;
light induced D-V patterned neuruloid: GSE163505;
chip-based neuruloid: GSE173492;
heart forming organoid: GSE150202;
somitoid: E-MTAB-11334.

Field-specific reporting

Please select the one below that is the best fit for your research. If you are not sure, read the appropriate sections before making your selection.

☒ Life sciences ☐ Behavioural & social sciences ☐ Ecological, evolutionary & environmental sciences

For a reference copy of the document with all sections, see nature.com/documents/nr-reporting-summary-flat.pdf

Life sciences study design

All studies must disclose on these points even when the disclosure is negative.

Sample size	The sample size of this study was determined based on availability of highly regulated primate embryo samples. In compliance with the 3R guidelines, we reduced the number of used animals to minimum and obtained pregnant females uteri at E20 (n=2), E22 (n=1), E23 (n=2), E26 (n=1), and E29 (n=1), which allowed us to obtain high coverage transcriptome for each cell type, and perform confident downstream analyses.
Data exclusions	First, single cells with a number of detected genes (nFeature_RNA) above 500 and detected transcripts (nCount_RNA) above 1000 were retained to exclude the apoptotic or dead cells. Then, the doublet or multiplet cells were figured out with the Scrublet, according to the recommended multiplet rate reference table from 10X Genomics (Wolock et al., 2019). Next, the Seurat objects of different samples were created independently with the expression matrix and metadata containing cell barcodes, cell status, and assignment information identified by Souporcell and cell multiplet information inferenced by Scrublet, then these Seurat3 objects were merged.
Replication	Sequenced samples from two independent embryos of the same stages showed similar gene expression patterns.<br>Since developmental stage of embryo in utero is uncontrollable, though we collected monkey embryos by calculating the day post fertilization and combining with b-ultrasound, CS10 embryo was not successfully collected, so not performed.<br>The IF experiments on mouse embryos were repeated in three independent biological samples.<br>The stem cells experiments were independently repeated at least three times. All attempts of experiment replication were successful.
Randomization	Samples were not allocated into randomized groups. Randomization was not relevant to the study. All embryo samples were analyzed individually.
Blinding	Blinding of the investigators was not possible due to study design and was not relevant to the study. It was not possible to blind the experiments during neither embryo collection nor single cell collection.<br>We performed lineage assignment in an unbiased way, in detail we assigned samples to lineages based on their gene expression profile and then validated our findings by their localization within the embryo.

Reporting for specific materials, systems and methods

We require information from authors about some types of materials, experimental systems and methods used in many studies. Here, indicate whether each material, system or method listed is relevant to your study. If you are not sure if a list item applies to your research, read the appropriate section before selecting a response.

Materials & experimental systems

n/a	Involved in the study	
☐	☒	Antibodies
☐	☒	Eukaryotic cell lines
☒	☐	Palaeontology and archaeology
☐	☒	Animals and other organisms
☒	☐	Human research participants
☒	☐	Clinical data
☒	☐	Dual use research of concern

Methods

n/a	Involved in the study	
☒	☐	ChIP-seq
☒	☐	Flow cytometry
☒	☐	MRI-based neuroimaging

Antibodies

Antibodies used

Primary antibodies used for IF staining of embryos and embryonic stem cells:
OCT4 (mouse, 1:100, Santa Cruz Biotechnology, sc5279, clone C-10)
SOX2 (mouse, 1:100, Santa Cruz Biotechnology, sc365823, clone E-4)
E-Cadherin (goat, 1:200, R&D Systems, AF748)
OTX2 (goat, 1:100, R&D Systems, AF1979)
Slug (rabbit, 1:100, Cell Signaling Technology, 9585S, clone C19G7)
FOXA2 (rabbit, 1:200, Abcam, ab108422, clone EPR4466)
T (rabbit, 1:100, Cell Signaling Technology, 81694, clone D2Z3J)
AF9, MLLT3 (rabbit, 1:200, Abcam, ab154492)
Brachyury, T (goat, 1:200, R&D Systems, AF2085)
FOSB (goat, 1:200, R&D Systems, AF2214)
TBX6 (goat, 1:200, R&D Systems, AF4744)
YAP1 (mouse, 1:200, Abnova, H00010413-M01, clone 2F12)
EOMES (rabbit, 1:100, Abcam, ab23345)
CDX2 (mouse, 1:100, BioGenex, MU392A-5UC, clone CDX2-88)
GATA6 (goat, 1:100, R&D Systems, AF1700)
PDGF Receptor α (rabbit, 1:1, Cell Signaling Technology, 3174T, clone D1E1E)

Secondary antibodies used for IF staining of embryos and embryonic stem cells:
Alexa Fluor 488 Donkey anti-mouse (1:200, Thermo Fisher, A21202)
Alexa Fluor 647 Goat anti-mouse (1:200, Thermo Fisher, A31571)
Alexa Fluor 568 Donkey anti-rabbit (1:200, Thermo Fisher, A10042)
Alexa Fluor 647 Donkey anti-rabbit (1:200, Thermo Fisher, A31573)
Alexa Fluor 488 Donkey anti-goat (1:200, Thermo Fisher, A11055)
Alexa Fluor 488 Donkey anti-rat (1:200, Thermo Fisher, A21208)
Alexa Fluor 568 Donkey anti-sheep (1:200, Thermo Fisher, A21099)

Validation

All the above primary antibodies were validated in this work, the subcellular localization of the analyzed proteins has been previously reported. We used this to validate the specificity of the antibody in this study.
Primary antibodies used for IF staining of embryos and embryonic stem cells:
SOX2 (mouse, 1:100, Santa Cruz Biotechnology, sc365823, clone E-4): Correctly stained on a E22 monkey embryo, embryonic day (E)7.5-E8.5 mouse embryos for ectoderm as expected, and previously reported (PeerJ (2019), doi: 10.7717/peerj.5840 in porcine blastocyst).
SOX2 (rabbit, 1:200, Cell Signaling Technology, 5024S, clone D6D9): Correctly stained on human, monkey, and mouse ESC-derived neuromesodermal progenitor (NMP)-like cells for in vitro differentiation as expected, and previously reported (PLoS ONE (2014), doi: 10.1371/journal.pone.0106694 in human fetal normal brain sections and glioblastoma patient derived cell lines).
E-Cadherin (goat, 1:200, R&D Systems, AF748): Correctly stained a E22 monkey embryo for epithelial cells as expected, and previously reported (Developmental Cell (2017), doi: 10.1016/j.devcel.2017.05.004 in mouse ESCs).
OTX2 (goat, 1:100, R&D Systems, AF1979): Correctly stained a E22 monkey embryo and E7.5 mouse embryo for visceral endoderm, definitive endoderm, neural ectoderm and some mesoderm, as expected, and previously reported (Development (2018), doi: 10.1242/dev.167833 in monkey and human late blastocysts).
SLUG (rabbit, 1:100, Cell Signaling Technology, 9585S, clone C19G7): Correctly stained a E22 monkey embryo for epithelial-mesenchymal transition as expected, and previously reported (Scientific Reports (2018), doi: 10.1038/s41598-018-30939-z in human ESCs).
FOXA2 (rabbit, 1:200, Abcam, ab108422, clone EPR4466): Correctly stained a E22 monkey embryo and E7.5 mouse embryo for endoderm and some mesoderm, as expected, and previously reported (BioRxiv (2022), doi: 10.1101/2022.03.07.483315 in E7.5 mouse embryos).
T (rabbit, 1:200, Cell Signaling Technology, 81694, clone D2Z3J): Correctly stained a E22 monkey embryo and E7.5 mouse embryo, and E8.5 mouse embryos for primitive streak (PS) and some mesoderm, as expected, and previously reported (BioRxiv (2022), doi: 10.1101/2022.03.07.483315 in E7.5 mouse embryos).
Correctly stained on human, monkey, and mouse ESC-derived NMP-like cells and presomitic mesoderm (PSM)-like cells for in vitro differentiation as expected, and previously reported (BioRxiv Preprint (2022), doi: 10.1101/2022.03.07.483315 in E7.5 mouse embryos).
Brachyury (T) (goat, 1:300, R&D Systems, AF2085-SP): Correctly stained on human, monkey, and mouse ESC-derived NMP-like cells and presomitic mesoderm (PSM)-like cells for in vitro differentiation as expected, and previously reported (eLife (2018), doi: 10.7554/eLife.38279 in human ESCs).
AF9, MLLT3 (rabbit, 1:200, Abcam, ab154492): Correctly stained on monkey and mouse ESC-derived PSM-like cells for in vitro differentiation as expected, and previously reported (Transcription (2017), doi: 10.1080/21541264.2017.1295831 in HEK-293T cells).
FOSB (goat, 1:200, R&D Systems, AF2214): Correctly stained on human, monkey and mouse PSM-like cells for in vitro differentiation as expected, and previously reported (Molecular Systems Biology (2021), doi: 10.15252/msb.202010125 in U937 cells for western blot).
TBX6 (goat, 1:200, R&D Systems, AF4744): Correctly stained a E22 monkey embryo and E7.5 mouse embryo, and E8.5 mouse embryos for PS, NMP, and some mesoderm, as expected, and previously reported (Development (2018), doi: 10.1242/dev.164319 in mouse embryos between E9.5-10.5).
Correctly stained human, monkey and mouse ESC-derived PSM-like cells for evaluation of PSM-like cell differentiation as expected, and previously reported (Cell Reports (2019), doi: 10.1016/j.celrep.2019.07.090 in human ESCs-differentiated PSM cells).
YAP1 (rabbit, 1:200, Abcam, ab52771, clone EP1674Y): Correctly stained human, monkey and mouse ESC-derived PSM-like cells for Hippo-YAP activities, as expected, and previously reported (Oncology Reports (2019), doi: 10.3892/or.2019.7065 in human glioblastoma cells U-372 MG cells).
EOMES (rabbit, 1:100, Abcam, ab23345): Correctly stained a E22 monkey embryo, a E7.5 mouse embryo, and E8.5 mouse embryos for PS as expected, and previously reported (PLoS Biology (2020), doi: 10.1371/journal.pbio.3000705 in organoid models consisting of human ESCs and induced pluripotent stem cells).

CDX2 (mouse, 1:100, BioGenex, MU392A-5UC, clone CDX2-88): Correctly stained a E22 monkey embryo, E7.5-E8.5 mouse embryos for PS as expected, and previously reported (Developmental Cell (2017), doi: 10.1016/j.devcel.2017.05.004 in E3.5 mouse blastocysts).
GATA6 (goat, 1:100, R&D Systems, AF1700): Correctly stained a E22 monkey embryo for extraembryonic mesenchymal cells, endoderm and some mesoderm, as expected, and previously reported (Development (2018), doi: 10.1242/dev.167833 in monkey and human late blastocysts).
PDGF Receptor α (rabbit, 1:1, Cell Signaling Technology, 3174T, clone D1E1E): Correctly stained a E22 monkey embryo for mesoderm as expected, and previously reported (Disease Models & Mechanisms (2013), doi: 10.1242/dmm.013748 in E13.5 mouse embryo head skin sections).

Eukaryotic cell lines

Policy information about cell lines

Cell line source(s)	Human embryonic stem cell line H9 (WA09) was obtained from WiCell and authenticated by short tandem repeat (STR) profiling. Mouse epiblast stem cells (EpiSCs), rhesus macaque ES cells (ESCs) were generated and described in previous study (Wu, J. et al., Cell, 2017; Wu, J. et al., Nature, 2015). HES7 promoter-luciferase reporter line was generated by following a protocol described in the previous study (Matsuda, M. et al., Nature, 2020).
Authentication	Human embryonic stem cells (H9) were obtained from WiCell and authenticated by short tandem repeat (STR) profiling. Monkey and mouse cell lines were validated by IF staining, western blots using specific antibodies and genomic PCR and qRT-PCR using species-specific primers.
Mycoplasma contamination	All cell lines are negative for mycoplasma contamination.
Commonly misidentified lines (See ICLAC register)	No commonly misidentified cell lines were used in this study.

Animals and other organisms

Policy information about studies involving animals; ARRIVE guidelines recommended for reporting animal research

Laboratory animals	Male and female common cynomolgus monkeys (Macaca fascicularis) with an age range of 6-8 years old were used in this study. Male and female C57BL/6 mouse (Mus musculus) around 8 weeks old were used in this study.
Wild animals	This study did not use any wild animals.
Field-collected samples	This study did not use any field-collected samples.
Ethics oversight	This study was conducted in accordance with the “Principles for the Ethical Treatment of Non-Human Primates” issued by Institute of Zoology, Chinese Academy of Sciences (IOZ, CAS), and was approved in advance by the Institutional Animal Care and Use Committee of the IOZ, CAS (Appl.No: IOZ-EU-20191113 for all monkey experiments, Appl.No: IOZ-IACUC-2021-037 for all mouse experiments). Both followed relevant guidelines and regulations.

Note that full information on the approval of the study protocol must also be provided in the manuscript.
